# Therapeutic potential of gene therapy for gastrointestinal diseases: Advancements and future perspectives

**DOI:** 10.1016/j.omto.2023.08.007

**Published:** 2023-08-18

**Authors:** Ning-ning Yue, Hao-ming Xu, Jing Xu, Min-zheng Zhu, Yuan Zhang, Cheng-Mei Tian, Yu-qiang Nie, Jun Yao, Yu-jie Liang, De-feng Li, Li-sheng Wang

**Affiliations:** 1 Department of Gastroenterology, Shenzhen People’s Hospital (the Second Clinical Medical College, Jinan University), Shenzhen 518000, China; 2 Department of Gastroenterology and Hepatology, Guangzhou Digestive Disease Center, Guangzhou First People’s Hospital, School of Medicine, South China University of Technology, Guangzhou 510000, China; 3 Department of Gastroenterology and Hepatology, The Second Affiliated Hospital, School of Medicine, South China University of Technology, Guangzhou 510000, China; 4 Department of Medical Administration, Huizhou Institute of Occupational Diseases Control and Prevention, Huizhou, Guangdong 516000, China; 5 Department of Emergency, Shenzhen People’s Hospital (the Second Clinical Medical College, Jinan University, the First Affiliated Hospital, Southern University of Science and Technology), Shenzhen 518000, China; 6 Department of Gastroenterology, Shenzhen People’s Hospital (the Second Clinical Medical College, Jinan University, the First Affiliated Hospital, Southern University of Science and Technology), Shenzhen 518000, China; 7 Department of Child and Adolescent Psychiatry, Shenzhen Kangning Hospital, Shenzhen 518000, China

**Keywords:** gene therapy, gastrointestinal disease, gene therapy targets, delivery carriers, clinical trials

## Abstract

Advancements in understanding the pathogenesis mechanisms underlying gastrointestinal diseases, encompassing inflammatory bowel disease, gastrointestinal cancer, and gastroesophageal reflux disease, have led to the identification of numerous novel therapeutic targets. These discoveries have opened up exciting possibilities for developing gene therapy strategies to treat gastrointestinal diseases. These strategies include gene replacement, gene enhancement, gene overexpression, gene function blocking, and transgenic somatic cell transplantation. In this review, we introduce the important gene therapy targets and targeted delivery systems within the field of gastroenterology. Furthermore, we provide a comprehensive overview of recent progress in gene therapy related to gastrointestinal disorders and shed light on the application of innovative gene-editing technologies in treating these conditions. These developments are fueling a revolution in the management of gastrointestinal diseases. Ultimately, we discuss the current challenges (particularly regarding safety, oral efficacy, and cost) and explore potential future directions for implementing gene therapy in the clinical settings for gastrointestinal diseases.

## Graphical abstract

**Figure:**
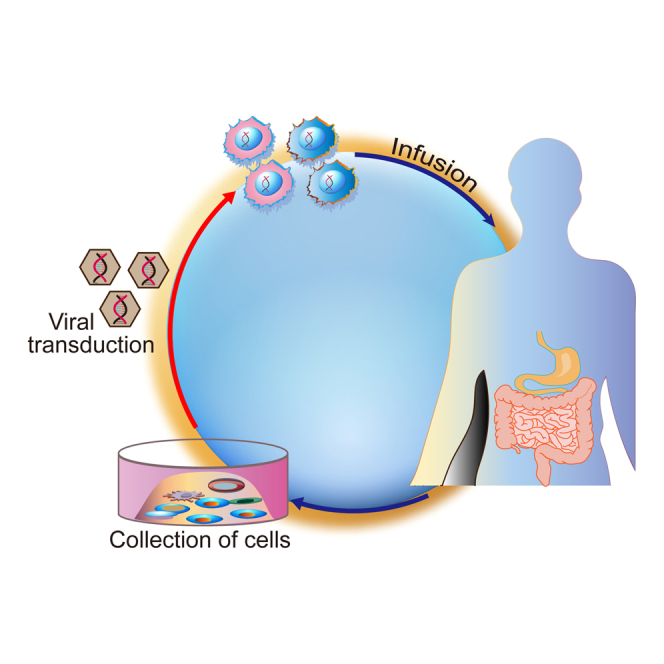

## Teaser

Wang and colleagues review the key gene therapy targets and delivery systems in gastroenterology, fueling the revolution of gene therapy in the treatment of gastrointestinal disorders. Moreover, they summarize recent advances in gene therapy for gastrointestinal disorders and the challenges faced in the clinical implementation of this approach.

## Introduction

Genetic abnormalities have been associated with various gastrointestinal (GI) and liver diseases, including digestive tumors, inflammatory bowel disease (IBD), gastroesophageal reflux disease, pancreatitis, and non-alcoholic fatty liver disease, as well as irritable bowel syndrome.^1^^,^^2^^,^^3^^,^^4^^,^^5^ With advancements in vector delivery and gene-editing techniques, gene therapy provides a promising approach for treating GI diseases that cannot be fixed by conventional pharmaceuticals and surgeries. There are four main therapeutic strategies in gene therapy, namely gene addition, gene editing, mRNA therapy, and gene silencing.^6^

**Figure 1:**
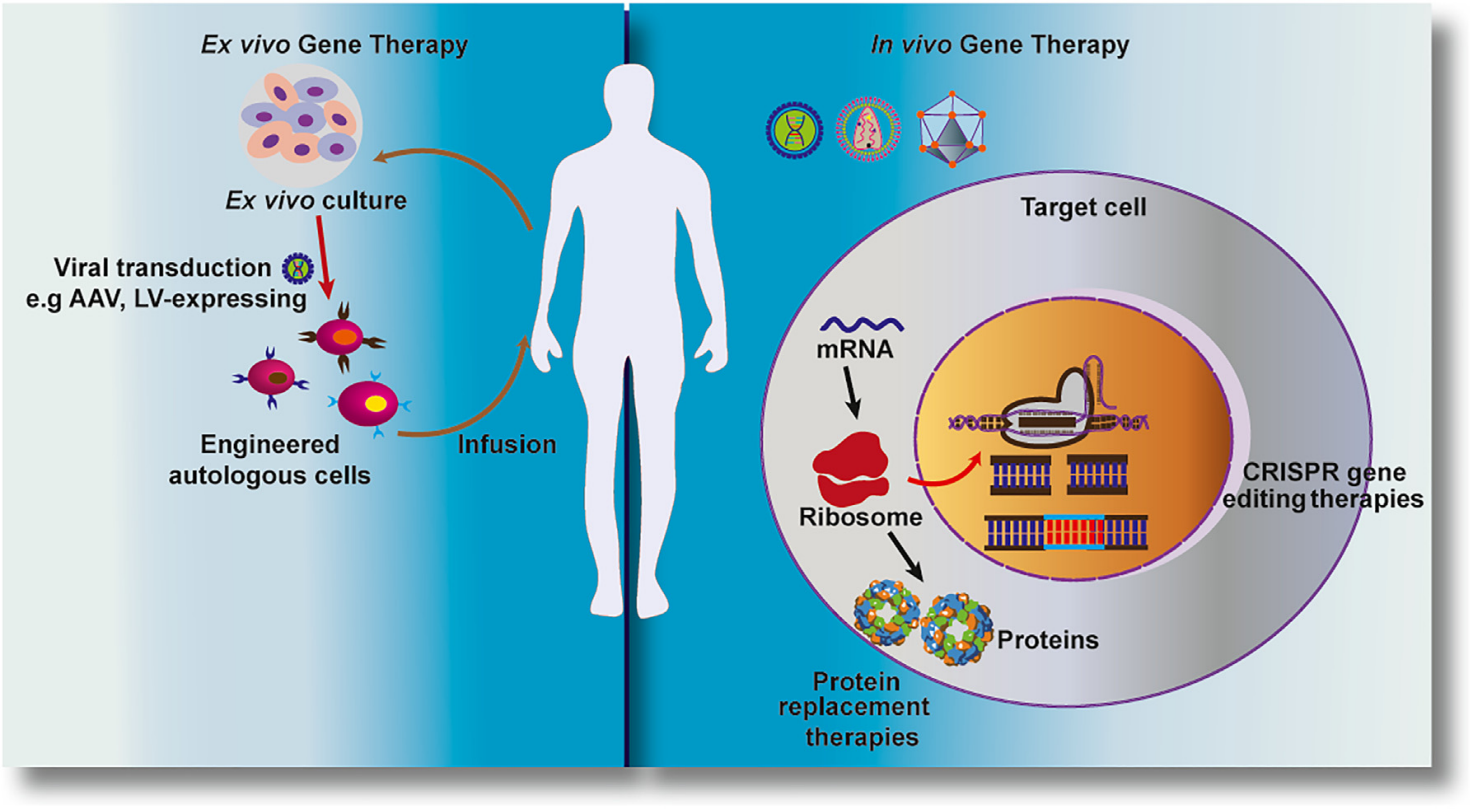
Schematic diagram of *ex vivo and in vivo* strategies for gene therapy *Ex vivo* gene therapy starts with the extraction of autologous stem cells into which the gene is transferred, then these cells will be reintroduced into the body by intravenous injection, where the stem cells can differentiate and the target gene can be expressed. *In vivo* gene therapy involves introducing the therapeutic gene directly into the viral genome and then transducing the target cells by intravenous injection to overexpress the therapeutic gene or correct the pathological gene.

Gene therapy involves the transduction of exogenous normal genes into impacted cells or organisms to rectify or compensate for pathogenic genes. It holds therapeutic potential for terminal or severely debilitating diseases. The goal is to achieve sustained expression of therapeutic genes at levels adequate to improve or cure disease manifestations while minimizing adverse effects.^6^ Therapeutic genes materials for diseases can include plasmid DNA (pDNA), mRNA, small interfering RNA (siRNA), microRNA (miRNA), and short hairpin RNA (shRNA). These gene materials can be rapidly and precisely modified using gene-editing techniques, with CRISPR-Cas9 being a pioneering method.^7^ Current gene therapy strategy primarily revolves around two approaches: *ex vivo* and *in vivo* (Figure 1). *Ex vivo* transduction involves isolating target cells from the patient, introducing the therapeutic gene into these cells, modifying the cell gene, and then returning the modified cells to the patient for disease treatment, like the chimeric antigen receptor T (CAR-T) cell treatment. This approach typically requires target genes, delivery carriers with integration capabilities, and sophisticated techniques for cell manipulation. In contrast, the *in vivo* route involves directly delivering a gene into the patient’s body via a suitable delivery vector. This approach avoids the multistep *ex vivo* process, which is similar to the straightforward delivery of conventional pharmaceuticals. In this case, the introduced gene does not integrate into the cellular genome but functions as an additional gene. The targeted cells in this approach are usually long-lived postmitotic cells that no longer divide, enabling long-term gene expression as long as the introduced DNA remains stable in the cells.^6^ Currently, gene-delivery vehicles comprise both viral and non-viral carriers. Viral vectors, including retroviruses, adeno-associated viruses (AAVs), adenoviruses (Ads), and lentiviruses, possess natural infectivity toward cells.^8^ Non-viral vectors are also rapidly developing and mainly include cationic polymorphic vectors, liposomal vectors, hydrogel vectors, and others. Non-viral vectors are more attractive from a safety standpoint, although further work is needed to enhance their transfection efficiency.^9^

**Figure 2:**
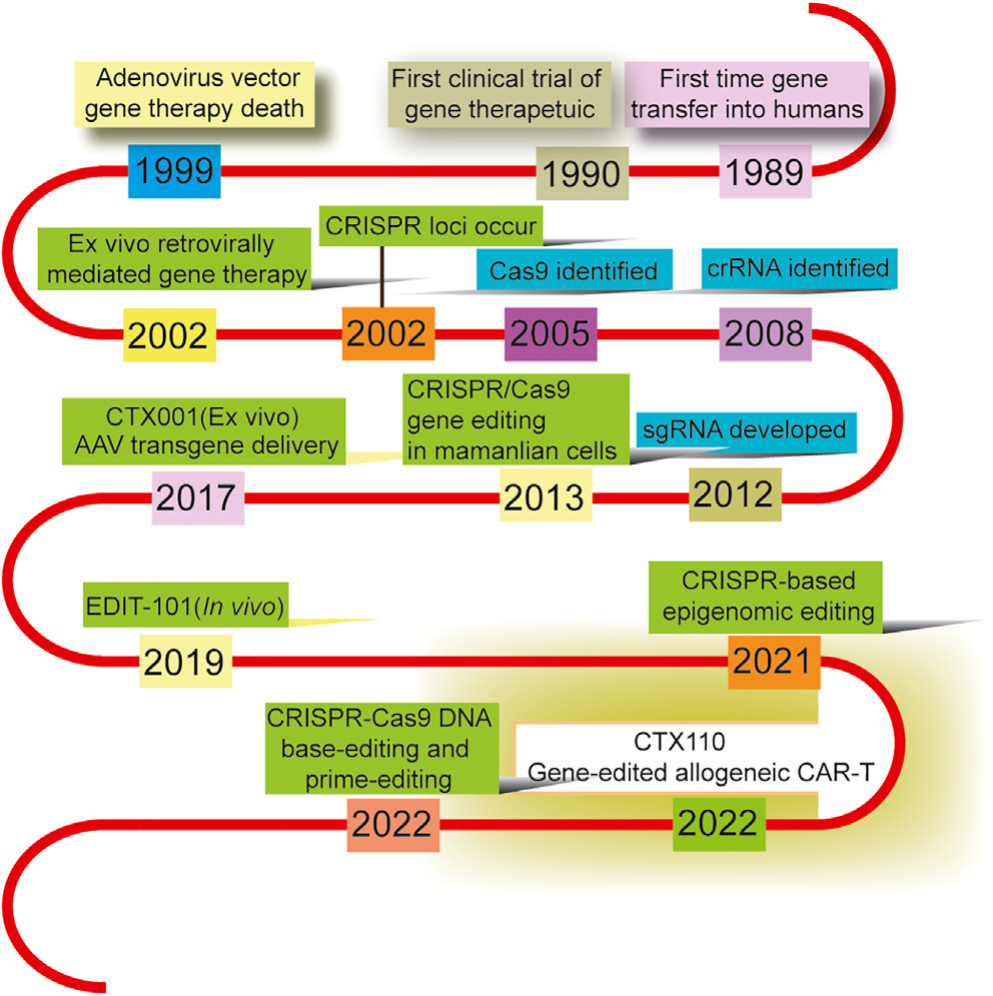
Timeline of milestones in the development of gene therapy technologies Gene therapy methods and their year of approval are shown above each milestone, along with the treatments being investigated.

Since the first clinical study involving gene therapy for a rare immunodeficiency disorder in 1990,^10^ significant progress has been made in the field. Gene-editing treatments, in particular, have received approval over the years. The approved therapies and the year of approval are listed below each milestone in Figure 2, along with alternative therapies being explored. At present, there have been approximately 17 nucleic acid products approved globally, and nearly 2,700 clinical trials have been completed, are ongoing, or have received approval. These trials cover a broad range of applications, including monogenic diseases, infectious diseases, cardiac disorders, neurological diseases, and GI diseases.^11^^,^^12^^,^^13^^,^^14^^,^^15^ In recent years, with an improved understanding of the pathogenesis of digestive diseases, it seems likely that more gene therapies can be identified and designed. Moreover, the GI tract presents a unique opportunity for gene drug administration through various delivery methods, such as oral, endoscopic, and rectal routes. This characteristic makes gene therapy highly appealing for treating GI conditions.

Here, we summarize the targets and regulated signaling pathways of gene therapy in GI diseases as well as the applications of targeted delivery systems. We also give an overview of the advancements in preclinical and clinical studies on gene therapy for digestive diseases. Finally, we discuss the existing challenges associated with clinical applications and propose potential future research directions.

## Target gene for GI diseases

The success of gene therapy in treating GI ailments relies on the identification of gene targets associated with each disease, which necessitates a deep understanding of their molecular biology. Given the diverse etiology and severity of GI diseases, as well as the presence of multiple gene alterations in GI malignancies, selecting appropriate target genes presents the most significant challenge. We categorize and summarize the key gene targets and signaling pathways in gene therapy for GI illnesses based on their respective gene functions.

### Tumor-suppressor genes

p53, an anti-oncogene located on the short arm of chromosome 17 in humans, encodes a tumor-suppressor protein that functions as a transcription factor. It plays a crucial role in regulating cell-cycle initiation, DNA repair, and apoptosis by interacting with various gene-specific sequences, such as p21, Bax, and Bcl-2.^16^ In healthy cells, p53 levels are kept low through polyubiquitination of the E3 ubiquitin ligase MDM2. However, in stressed cells, such as those experiencing DNA damage or oncogenic stress responses, the interaction between p53 and MDM2 is interrupted, leading to the stabilization and activation of p53 to carry out its functions.^17^ Mutations in the p53 gene, often missense mutations, result in the loss of its tumor-suppressing function and promote the development and progression of various tumors, including those in the digestive system.^18^ In a phase 1 clinical trial of gene therapy for advanced solid tumors, liposomal nanoparticles are used as a delivery system to introduce p53 DNA into target cells. This intervention successfully restores the function of the tumor-suppressor gene p53, leading to significant suppression of solid tumor growth, including colorectal cancer (CRC).^19^

Another important gene target is the retinoblastoma (RB) gene, which acts as a transcriptional corepressor and plays a critical role in cell-cycle regulation. RB family proteins interact with the transcription factor E2F to inhibit gene transcription. Phosphorylation of RB proteins, typically by cyclin-dependent kinase (CDK)2/4/6, leads to their transformation into phosphorylated RB (p-RB), which releases E2F and activates cyclin D, encouraging DNA replication and the transition from the G1 phase to the S phase.^20^^,^^21^ Mutations in the RB gene result in loss of control over cell proliferation and impairment of cell-cycle checkpoint control, which are critical steps in tumor growth. The p16 gene, located on chromosome 9p21, is part of the INK4 family, which consists of p15 ^INK4B^, p16 ^INK4A^, p18 ^INK4C^, and p19 ^INK422^. p16 functions as a CDK inhibitor (CDKI) and is found to be mutated in 85% of pancreatic adenocarcinomas.^23^ In the cell cycle, p16 negatively regulates the pRb-E2F pathway.^24^ It causes hypophosphorylation of p-RB by binding to CDK4 and CDK6. Additionally, p16 can disrupt the complex formed by CDK6/4 and non-p16 inhibitors, thereby amplifying the effect of non-p16 inhibitors, decreasing CDK2 activity, and elevating hypophosphorylated p-RB, which results in cell-cycle arrest.^22^ After infecting p16-deficient laryngeal cancer Hep2 cells with recombinant Ads carrying the p16 gene, Zhang et al. observed a significant increase in p16 protein expression, accompanied by a marked reduction in cell proliferation, invasion, and tumor volume.^25^ Besides, the phosphatase and tensin homolog (PTEN) and SMAD signaling pathways are also prevalent tumor-suppressor genes in GI tumors.^26^^,^^27^ Taken together, enhancing the expression of these genes holds promise for slowing tumor progression.

### Oncogenes

The Ras gene, which contains H-Ras, K-Ras, coupled with N-Ras, is the most prevalent oncogene in malignancies, with K-Ras mutations found in almost all GI tumors. It is present in 35% of colon tumors and 95% of pancreatic tumors.^28^^,^^29^ The K-Ras gene encodes a kind of small GTPases and plays a role in modulating various cellular processes, such as cell growth, proliferation, apoptosis, and differentiation. Its activation is mediated by upstream signals, such as cell surface growth factor, cytokine, and hormone receptors.^28^^,^^30^ Mutations in the K-Ras gene result in dysfunctional GTPases, leading to the activation of MAPK, PI3K, and RAF signaling pathways, which are implicated in tumorigenesis and disease progression.^28^ Consequently, gene-silencing strategies that reduce the expression of proto-oncogenes and disrupt the signaling pathways driving carcinogenesis hold the potential for improving tumor progression.

### Suicide genes

Suicide gene therapy is a widely used treatment for solid malignancies. In this approach, a transgene encoding an enzyme is introduced into target cells, leading to the conversion of an inactive prodrug into a cytotoxic substance that selectively destroys tumor cells. This therapy can also enhance its efficacy through the bystander effect. Currently, several suicide genes are the focus of extensive research. Herpes simplex virus thymidine kinase/ganciclovir (HSV-TK/GCV) system represents the most commonly used suicide gene strategy. Transducing HSV-TK into tumor cells induces the expression of viral TK, which phosphorylates GCV into its triphosphorylated form. This activated GCV inhibits DNA polymerization and is integrated into synthesized DNA, causing base-pairing errors and DNA-chain breakages. Consequently, tumor cell division is arrested, and cell death occurs.^31^ Thus, HSV-TK enhances the sensitivity of the antiviral medication GCV to kill tumor cells. Another frequently employed suicide gene regimen is the cytosine deaminase/5-fluorocytosine (CD/5-FC) system. The CD gene encodes CD, which converts the non-toxic prodrug 5-FC into the cytotoxic 5-fluorouracil (5-FU). The CD/5-FC system inhibits cell proliferation and induces cell death, making it a standard chemotherapy drug for hepatocellular carcinoma (HCC) management.^32^ Furthermore, the purine nucleoside phosphorylase/6-methylpurine deoxyribose (PNP/MePdR) system has shown beneficial anti-tumor effects in pancreatic cancer. PNP converts the non-toxic prodrug MePdR into the cytotoxic compound 6-methylpurine (MeP) with a significant bystander effect, selectively killing tumor cells.^33^ Other suicide gene therapies have also been introduced into oncology treatment, including inducible caspase-9 (iCasp9), varicella-zoster virus TK (VZV-TK), and nitroreductase.^34^^,^^35^ However, the precise mechanisms of action for these suicide genes have not been thoroughly elucidated.

### Angiogenesis-related genes

In gene therapy for digestive illnesses, there is great anticipation for therapeutic approaches targeting angiogenesis genes. The vascular endothelial growth factor (VEGF) signaling pathway serves an important role in tumor vasculogenesis. The VEGF gene encodes the VEGF protein family, comprising VEGF-A, VEGF-B, VEGF-C, VEGF-D, and VEGF-E, as well as placental growth factor. These proteins bind to VEGF receptors on endothelial cells, activating downstream signaling pathways, including Ras/MAPK, FAK, IP3, Akt/PKB, and PI3K/Akt/mTOR. These pathways regulate cell survival, proliferation, migration, and permeability, thereby maintaining angiogenesis homeostasis.^36^^,^^37^ In the majority of GI tumors, there is an increase in the expression of genes related to the VEGF signaling pathway. This upregulation facilitates tumor progression by promoting endothelial cell survival, vascular abnormalization, neovascular growth, and vascular permeability.^38^^,^^39^ Hence, targeting gene expression in the VEGF signaling pathway through knockdown strategies, utilizing materials such as siRNAs, antisense oligonucleotides (ASOs), and ribozymes, holds promise for anti-angiogenic therapy in tumors. On the other hand, in peptic ulcerative diseases, overexpression of VEGF genes can stimulate angiogenesis and dramatically accelerate the healing of ulcer mucosal.^40^

### Immune-related genes

Gene therapy associated with the immune system represents an innovative and promising strategy for managing digestive disorders. Interleukin-12 (IL-12) has emerged as a significant immunomodulatory factor with potent anti-tumor activity. IL-12, composed of the P40 and P35 subunits, is primarily produced by macrophages and dendritic cells.^41^ The binding of IL-12 to the IL-12 receptor activates natural killer cells (NKs) and T cells, leading to the production of interferon γ (IFN-γ). IL-12 and IFN-γ improve the cytotoxic effects of CD8^+^ T cells, resulting in the generation of perforin and granzyme, coupled with Fas ligand (FasL), which mediate tumor-suppressive effects.^42^^,^^43^ Moreover, IFN-γ suppresses the expression of VEGF and matrix metalloproteinase-9 (MMP-9) in bone marrow cells, thereby inhibiting angiogenesis.^44^ Other members of the IL-12 family, such as IL-23 and IL-27, as well as IL-35, also exert a crucial function in tumor immunomodulatory.^41^ Therefore, IL-12 holds potential as a candidate for gene therapy in GI tumors. Besides, CAR-T cell therapy has demonstrated strong efficacy in terms of anti-tumor activity. Genetically modified T cells express chimeric receptors that specifically recognize antigens on tumor cells, enhancing cytokine and IL production and promoting anti-tumor immune responses. The targets of CAR-T cells in GI tumors mainly include CEA, Claudin18.2, CD133, CD28, MUC-1, HER-2, Glypican-3 (GPC3), GUCY2C, and epidermal growth factor receptor (EGFR).^45^^,^^46^^,^^47^^,^^48^^,^^49^ On the other hand, IL-10, an immunosuppressive factor, negatively regulates proinflammatory factors and possesses the ability to limit immunity. IL-10 primarily acts on antigen-presenting cells (APCs), such as macrophages and monocytes, to suppress the production of proinflammatory cytokines, including tumor necrosis factor α (TNF-α), IL-1β, and IL-6, coupled with IL-8.^50^ Additionally, IL-10 inhibits key factors involved in CD4^+^ T cell differentiation, such as IL-12 and IL-23.^51^^,^^52^ Furthermore, it directly influences T cells, limiting their proliferation and cytokine production.^53^ IL-10 mutations are related to vulnerability to IBD.^54^ Definitively, mice with IL-10 gene deletion can develop spontaneous colitis.^55^ Therefore, enhancing IL-10 gene expression is expected to limit the inflammatory response in IBD.

### Other disease-associated genes

Investigations into the pathophysiology of IBD have uncovered the significant role of specific gene mutations. Among these mutations, autophagy-related 16-like 1 (ATG16L-1) as well as nucleotide-binding oligomerization domain containing 2 (NOD2) have been identified as particularly influential in increasing susceptibility to IBD. NOD2, positioned on the long arm of human chromosome 16, encodes the NOD2 protein, a peptidoglycan-derived intracellular pattern recognition receptor for bacteria. It plays a crucial role in maintaining mucosal homeostasis and inducing mucosal inflammation.^56^ Mutations in the NOD2 gene primarily increase susceptibility to Crohn’s disease, weakening the body’s first line of defense against infection and dysregulating the nuclear factor κB (NF-κB) signaling pathway, resulting in mucosal inflammation.^57^ The ATG16L-1 gene is involved in cellular autophagy, coding polymorphism (T300A). Genomic variations in ATG16L-1 T300A lead to increased degradation of the ATG16L-1 T300A protein by caspase-3 and caspase-7. This can cause defective autophagy, leading to restricted endoplasmic reticulum (ER) stress in Paneth cells, diminished anti-microbial effects, and increased IL-1β levels. These mechanisms are involved in the development of Crohn’s disease.^58^ In addition, covalently closed circular DNA (cccDNA) is established by hepatitis B virus (HBV) in the nucleus of infected cells. This is a primary reason for the failure to eradicate HBV. cccDNA is the original replication template for HBV pregenomic RNA and is extremely stable.^59^ Consequently, eliminating cccDNA from the host cell nucleus by gene-silencing strategies may contribute to a complete cure for hepatitis B. Moreover, there are other rare diseases related to gene mutations that affect the digestive system, such as the canalicular membrane protein ATP-binding cassette subfamily B member 4 (ABCB4) mutations in progressive familial intrahepatic cholestasis type 3 (PFIC3),^60^ JAG1 gene or receptor NOTCH2 mutations in Alagille syndrome,^61^ ATP7B gene mutations in Wilson disease,^62^ and SERPINA1 gene mutations in Alpha-1 anti-trypsin deficiency.^63^ Currently, there are limited therapeutic options for these rare conditions, and liver transplantation is often considered a last resort. Hence, correcting the phenotypes caused by genetic abnormalities by appropriate gene therapy strategies provides a promising alternative therapeutic approach for the treatment of rare diseases.

## Gene delivery to GI tract

### AAV-based vectors for gene therapy

Since the discovery of AAVs in 1965, they have greatly fueled interest in gene therapy research for various diseases, owing to their high transfection rate, extensive host cells (dividing and non-dividing cells), good security, and persistent expression of exogenous genes.^64^^,^^65^ Belonging to the genus *Dependoparvovirus* of the family *Parvoviridae*, AAVs are defective viruses that rely on helper viruses (such as Ads or herpesviruses) for replication. This feature makes them attractive as gene-delivery vectors for transfecting target cells. AAVs consist of an icosahedral protein capsid (∼20–26 nm) and a linear single-stranded DNA (4.7 kb) that can be either sense or antisense.^66^

To eliminate the risk of insertional mutagenesis, recombinant AAVs (rAAVs) have been designed. rAAVs, modified from wild-type AAVs, retain the same capsids as wild-type AAVs but have their rep and cap genes replaced with targeted genes. They retain only the two inverted terminal repeats (ITRs) at each end, eliminating their ability to integrate into the host genome.^65^ rAAVs exhibit a broad tropism for infecting both dividing and quiescent cells, making them highly advantageous for gene therapy of liver diseases. Moreover, rAAV vectors have prolonged expression durations and lower immunogenicity compared with adenoviral vectors.^67^ Currently, AAVs are roughly classified into 13 serotypes (AAV1–AAV13) according to the amino acid sequences of their capsid proteins.^68^ The interaction between AAVs and cell membrane receptors is the initial step in infection, and different serotypes exhibit varying tissue affinities due to differences in cell membrane composition. Therefore, the selection of the appropriate serotypes is crucial for the successful gene delivery. Although AAV2 is the most widely used serotype in digestive diseases, multiple studies have proved that AAV8 outperforms AAV2 and other serotypes in terms of hepatic transgene expression.^69^ Extensive preclinical and clinical research has been conducted to identify the optimal AAV serotype for GI and liver tissue. Polyak et al., for instance, have evaluated the efficiency of rAAV-mediated gene transduction in intestinal epithelial cells both *in vivo* and *ex vivo*, demonstrating that rAAV2 more efficiently transduces human colonic epithelial cells *ex vivo*. Transgenic assays conducted 80 days after AAV treatment reveal successful transduction of crypt progenitor cells, suggesting the feasibility of AAV-mediated gene delivery in the gut.^70^ In 2022, Ma et al. have explored the efficacy of AAV9-mediated colon transduction through intraperitoneal injection, demonstrating successful transduction of the colonic mucosa and submucosa in rats.^71^ Besides, Vilà et al. have used AAV8 to deliver the Sirt1 gene to the liver, resulting in long-term sustained expression of the Sirt1 gene and successfully preventing high-carbohydrate-diet-induced non-alcoholic fatty liver disease.^72^ Thus, AAV vectors have greatly facilitated the rapid development of gene therapy, propelling it to the forefront of therapeutic strategies for GI disorders.

However, AAVs as gene-delivery vectors also have several limitations and drawbacks that need to be overcome. Firstly, the loading capacity of AAV vectors is limited to approximately 4.7 kb exogenous DNA fragments. Therefore, it is necessary to expand the AAV vector volume in order to accommodate larger genes. Secondly, the host immune response poses a hurdle to AAV vector-based gene therapy both in terms of humoral and cellular immunity. This immune response can hinder repeated administration of the viral vector, thus limiting its clinical applications.^73^ Careful design of the viral capsid and therapeutic gene can help mitigate the immune response. Additionally, although the risk of insertional mutagenesis is low in rAAV, if insertions occur in oncogene hotpots, it can potentially lead to HCC.^74^ Finally, the prohibitive expense of large-scale production of AAV vectors for clinical use remains a tremendous challenge. To address these issues, efforts are being made to develop and design capsids with improved characteristics, expand vector capacity, and enhance packaging efficiency, transduction efficiency, and gene expression efficiency. Recent studies have shown that AAV vectors can be isolated from the conditioned medium of packaged cells by utilizing their binding capacity to exosomes (exo-AAV). Exo-AAV has demonstrated increased resistance to neutralizing anti-AAV antibodies compared with standard AAV. In contrast to the classical AAV iodixanol gradient purification protocol, the exo-AAV purification protocol relies only on the step of ultracentrifugation to separate the cell culture supernatant. Importantly, no cytotoxicity has been found in exosomal AAV-transduced cells.^75^ This highlights the potential of exogenous AAV as a novel pathway for gene delivery.

### Exosome-mediated gene therapy

Exosomes are a subgroup of lipid bilayer-coated nanovesicles that originate as intraluminal vesicles (ILVs) in late endosomal. They can be released by all cell types and serve as a natural carrier for intercellular communication. This function has inspirited researchers to develop exosome-based drug-delivery systems. Exosomes act as multifunctional carriers capable of encapsulating various gene therapy molecules, such as mRNA, miRNA, and DNA.^76^ Due to their nanoscale properties, exosomes can cross the blood-brain barrier to reach brain tissues. They can also migrate to tissues without a blood supply, such as dense connective tissue.^77^^,^^78^^,^^79^ Moreover, exosomes exhibit high cellular uptake efficiency, and their surface membrane proteins, such as tetraspanin and fibronectin, allow for easy modification to achieve selective targeting of specific tissues and cells. This enables precise gene therapy, preventing unnecessary accumulation in other organs.^80^ Plant-derived exosomes or bacterial outer membrane vesicles can even enter the intestinal tissues through oral administration, providing novel gene-delivery systems for the treatment of IBD.^81^^,^^82^

### Nanoparticle-based: Lipid-based particles/polymeric particles/inorganic particles

Although viral vectors have demonstrated efficient transduction rates, their limitations in nucleic acid loading capacity have prompted researchers to explore non-viral vectors. Nanoparticle-based vectors, facilitated by advancements in nanotechnology, have made great strides in gene delivery. Given their tiny size (10–100 nm), these nanoparticles effectively deliver therapeutic genes to target cells or tissues by engaging with the cell surfaces or intracellular biomolecules.^83^ Presently, employed nanoparticles for gene delivery include liposomes, polymers, and inorganic nanoparticles.^84^^,^^85^^,^^86^

In the 1970s, Bangham et al. established liposomes as efficient carriers for small-molecule medications and nucleic acids.^87^ Today, liposomes are among the most extensively studied non-viral vectors. As spherical vesicles, liposomes consist of a phospholipid bilayer with an aqueous phase core, typically composed of phosphatidylcholine, cholesterol, and lipids. Liposomes offer advantages such as low toxicity, minimal immunogenic response, and high nucleic acid loading.^88^ Cationic liposomes are capable of spontaneously binding and concentrating negatively charged DNA while maintaining its stability, forming complexes with a strong affinity for cell membranes. These nanostructured complexes, referred to as “liposome complexes,” enter the cell through endocytosis. Subsequent breakage by the endosomal membrane leads to the release of the target gene, which seems to be the main mechanism of liposomal gene delivery.^89^ Cationic liposomes can be easily synthesized and prepared, and various components (such as PEGylation and ligands) are added to improve cell-liposome interactions.^90^ Several studies have proven the efficiency and safety of this cationic liposome vector-based approach to gene delivery. Zhang et al., for instance, have successfully used cationic liposomes to deliver a protamine-IL-22-binding protein mRNA complex for colon cancer gene therapy, achieving high mRNA transport and expression efficiency.^91^ In addition, Peng et al. have employed cationic liposomes to transport SATB1 shRNA for gene therapy in gastric cancer, resulting in the successful inhibition of gastric cancer cell growth.^92^ However, permanently charged cationic liposomes tend to be cytotoxic and prone to rapid clearance by the reticuloendothelial system.^93^ To address this issue, ionizable cationic liposomes and neutral liposomes have been introduced. These liposomes protect nucleic acids from degradation during circulation, prolonging their half-life in the blood and facilitating greater accumulation in target tissues or organs. Furthermore, they enable the timely release of nucleic acids from target cells.^90^

Polymers constitute another attractive category of non-viral gene-delivery carriers due to their structural and functional diversity, which leads to increased transfection efficiency.^83^ Presently, polymeric non-viral vectors mainly include polyethyleneimine (PEI), poly-L-lysine (PLL), dendrimers, and biodegradable polymers.^88^^,^^94^ PEI remains the “gold standard” for measuring the efficiency of non-viral gene vectors.^88^ However, its high-molecular-weight branched form exhibits significant cytotoxicity and is not ideal for *in vivo* transfection. To mitigate its toxic effects, PEI has been coupled with non-ionic biocompatible polymers. For example, Zhao et al. have designed a series of low-toxicity glycopolymers/PEI complexes for gene delivery, demonstrating improved stability, enhanced transfection efficiency of pDNA delivery to hepatocytes, and lower cytoxicity compared with PEI/pDNA complexes.^95^ Intelligent polymers can be engineered with specific tissue targeting, as well as with chemical or physical stimulus sensitivity, and environmental responsiveness.^96^ Polymers as gene-delivery vehicles have been applied in various experiments on GI diseases. Wang et al. developed a novel nanocarrier, PEG-poly(amino acid), for the delivery of miRNA-139-5p, effectively inhibiting tumor growth and migration in CRC mice.^97^

Inorganic nanoparticles, primarily comprising gold nanoparticles, silica nanoparticles, and iron oxide nanoparticles, have gained attention as an emerging synthetic vector for the transport of nucleic acids. These nanoparticles offer tunable size, structure, and morphology, resulting in minimal cytotoxicity and optimal biocompatibility.^98^ Gold nanoparticles are particularly attractive for nucleic acid delivery owing to their distinctive optical characteristics and convenient synthesis, as well as their surface functionalization.^99^ Nucleic acids can be attached to gold nanoparticles by covalent or non-covalent interactions. Charged or hydrophobic groups and ligands can also be incorporated into the surface of gold nanoparticles to achieve specific binding to cell surface receptors, enabling highly effective and stable delivery of nucleic acids to target organs or to tissues with minimal cytotoxicity.^100^^,^^101^ Silica nanoparticles, on the other hand, offer numerous advantages for gene delivery, such as chemical inertness, thermal stability, tunable particle size, dual functional surfaces (cylindrical pore surface and external particle surface), extended cargo loading, and good biocompatibility.^102^ Mesoporous silica nanoparticles (MSNPs), characterized by their honeycomb shape, are widely used as silica carriers.^103^ Typically, nucleic acids are packed into MSNPs through weak non-covalent interactions. MSNPs are commonly modified with amination, metal cations, or cationic polymers to impart a net positive charge, enhancing electrostatic interactions with nucleic acids and increasing gene loading. However, the amount of cationic polymers needs to be carefully controlled to equilibrate the transduction efficiency and cytotoxicity for tuned MSNPs in gene delivery.^102^ Pore size and surface functionalization (such as the incorporation of various cationic macromolecules) significantly influence the loading capacity and release rate of nucleic acids. MSNPs with smaller pores enable the delivery of small nucleic acids with adjustable release rates, while larger pores allow for higher loading as well as faster release rates and protect genes from nucleases.^104^ Iron oxide nanoparticles have achieved successful results as delivery vehicles due to their high biocompatibility, surface-coating diversity, and superparamagnetic properties.^105^ Typically, anionic nucleic acids are combined with surface-engineered cationic iron oxide nanoparticles by electrostatic interactions and are selectively transported to the target location under an external magnetic field.^106^ Kim et al. have constituted a novel gene-delivery vector for magnetofection named PPMag, which consists of PEI-associated polycaprolactone (PCL)-superparamagnetic iron oxide nanoparticles (SPIONs). These polyplexes permit nucleic acid condensation and escape from endosome/lysosome after cellular internalization via the proton sponge effect. Compared with the PEI-SPION group, the PPMag group exhibits lower cytotoxicity and higher transfection efficiency.^107^ However, nanoparticle-based vectors face significant challenges in the complex *in vivo* environment, including extracellular barriers and cellular barriers. These challenges affect gene transfer efficiency, gene expression persistence, and long-term safety.

### Hydrogel-based vectors for gene therapy

Hydrogels constitute a class of polymers formed by an extremely hydrophilic three-dimensional lattice structure. They offer tunable physicochemical and biological, coupled with structural, features (such as stiffness, pore diameter, microstructure, degradability, stimulus response, etc.), making them highly suitable for a variety of biomedical applications.^108^^,^^109^ Hydrogels have been engineered to stimulus-responsive gene-delivery systems to regulate the release of nucleic acids and prolong gene expression. These systems can be controlled by various stimuli, including pH, temperature, magnetic/electric fields, ionic strength, inflammation, and external stress.^110^^,^^111^

There are two methods available to control the release of nucleic acid molecules loaded in hydrogels. The first involves the release of small molecules of nucleic acids similar to gel-coated materials. The second method involves the gradual decomposition of the polymeric matrix containing the nucleic acids, which is controlled by the biodegradation rate of the matrix material.^112^ Among hydrogel gene-delivery carriers, thermosensitive hydrogels, as “smart hydrogels,” have been the pioneer in the field due to their exceptional thermal reversibility, and their excellent gene-delivery capability has been confirmed by several studies. To cite an example, Zhao et al. employed N-acetylgalactosamine (GalNAc) to target the peptidyl-prolyl *cis*/*trans* isomerase (Pin1) gene in the liver. They employed cholesterol-modified anti-microbial peptide DP7 (DP7-C) as a vector for the treatment of HCC. The delivery of GalNAc-Pin1 siRNA using DP7-C and hydrogels greatly increased the stability and prolonged the silencing effect of Pin1 siRNA.^113^ Therefore, hydrogels offer promising potential as materials for gene delivery. However, unintended side effects of hydrogels (e.g., inflammation, fibrosis, and calcification) and long-term efficacy have limited their applications as gene vectors.^108^ These challenges can be addressed by optimizing the physicochemical properties of the hydrogel materials.

## Gene therapy for GI diseases

### Gene therapy for IBD

IBD is a complex disease caused by a dysregulated immune response involving intestinal microorganisms and influenced by environmental stressors in individuals with a genetic predisposition.^114^ Failure of existing therapeutic strategies (mainly immunosuppressive and immune-modulatory medications, as well as biological therapies), along with their associated adverse side effects, presents significant economic, social, and health challenges. The genetic component of IBD offers insights into the underlying pathogenesis, providing a prospective approach for targeting “undruggable” targets (Table 1). Recently, over 240 common genetic susceptibility loci associated with IBD have been identified, including 38 brand-new loci.^115^ Considerable expectation is placed on the mucosal immune homeostatic effects of cytokines, such as IL-10, known for their potent immunomodulatory activity. Numerous studies have provided compelling evidence for the potential of IL-10 gene therapy targeted at IBD mouse models. Evidence from an IBD mouse model has demonstrated that correcting the IL-10 receptor defect in macrophages is closely correlated with the therapeutic response. Transplanting wild-type macrophages into the *IL-10Rb*^−/−^ IBD mouse significantly ameliorates colitis symptoms.^116^ Moreover, Sasaki et al. administrated an adenoviral IL-10 vector to IBD mice through the intestine or peritoneum, resulting in a remarkable decrease in disease activity, prevention of weight loss, and protection against colon histopathologic injury.^117^

**Table 1:** List of different delivery materials and loading cargoes, therapeutic targets, and routes of administration for IBD relief

Delivery material	Target	Loading cargo	Characterization	Route of administration	Outcome	Reference
Lipid nanoparticles	IRF8	siRNA	S: 57.63 ± 3.2 Z: 0.7 ± 0.35	intravenous	silencing the IRF8 gene exerted powerful immunomodulatory effects in IBD	Veiga et al.^129^
	IL-10	mRNA	S: ∼63.7 ± 1.59 Z: 0.9 ± 0.28	intravenous	delivery of IL-10 mRNA significantly reduced the severity of colitis-related pathological symptoms and intestinal inflammation	Veiga et al.^198^
	Cyclin-D1	siRNA	S: ∼100 Z: ‒	intravenous	siRNA alleviated IBD by silencing the expression of cyclin D1	Peer et al.^199^
Polyethyleneimine-derived nanoparticles	TNF-α	siRNA	–	intravenous	siTNF-α combined with dexamethasone sodium phosphate induced efficient anti-inflammatory effects in IBD mice	Xu et al.^200^
	TNF-α	siRNA	S: 151.52 Z: 22.08	rectal	siTNF-α resulted in a significant reduction in TNF-α expression in IBD mice, accompanied by a marked improvement in intestinal inflammation	Frede et al.^201^
	CD98	siRNA	S: ∼480 Z: −5.26	oral	CD98 was downregulated in intestinal epithelial cells and intestinal macrophages, which effectively attenuated colitis	Laroui et al.^202^
	CD98	siRNA	S: 210 Z: +15	oral	siCD98 decreased the severity of colitis in mice	Xiao et al.^203^
	TNF-α	siRNA	S: 609 ± 37 Z: ‒	oral	the introduction of TNF-α siRNA effectively attenuated colitis in mice	Laroui et al.^204^
Chitosan-derived nanoparticles	TNF-α	siRNA	S: 245.60 ± 0.33 Z: +13.03 ± 0.65	oral	delivery of TNF-α siRNA effectively inhibited weight loss and MPO activity in mice with ulcerative colitis	Huang et al.^205^
	TNF-α	siRNA	S: 261.3 ± 5.6 Z: −6.3 ± 1.4	oral	codelivery of siTNF-α and recombinant human IL-22 could significantly inhibit inflammatory activity and promoted mucosal healing capacity	Xiao et al.^121^
	CD98	siRNA	S: 246.2 ± 7.8 Z: −13.7 ± 4.1	oral	codelivery of siCD98 and curcumin effectively protected the mucosal layer and reduced inflammation *ex vivo* and *in vivo*	Xiao et al.^206^
	TNF-α	siRNA	S: 143.3 ± 1.1 Z: +18.7 ± 0.6	oral	siRNA delivered by nanoparticles modified with a density of 4% mannose showed a stronger gene-silencing effect in IBD	Chu et al.^207^
	Map4k4	siRNA	S :147.2 ± 7.8 Z: +26.2 ± 2.0	oral	siMap4k4 significantly improved weight loss and colon length reduction in IBD mice	Zhang et al.^208^
Biodegradable polymers	SNX10	shRNA	–	oral	SNX10-shRNA were effective in reducing weight loss and alleviating intestinal mucosal injury and inflammatory infiltration in both acute and chronic IBD mice	Bao et al.^209^
	TNF-α	siRNA	S: 275.0 Z: ‒	*ex vivo*	siTNF-α effectively inhibited the expression and secretion of macrophage TNF-α *in vivo* and *ex vivo*, exerting a therapeutic effect on IBD	Xiao et al.^210^
Stimuli-responsive polymers	TNF-α	siRNA	S: 110–120 Z: ∼19	oral	enzyme- and PH-responsive nanogels loaded with TNF-α siRNA could effectively reduce TNF-α levels secreted by mouse macrophages	Knipe et al.^211^
	TNF-α	siRNA	S: ∼600 Z: ‒	oral	siTNF reduced the level of TNF-α mRNA in the colon and protected mice from ulcerative colitis	Wilson et al.^212^
Poly(amino acid)-derived nanoparticles	TACE	shRNA	S: 160 Z: 40	intravenous	shTACE effectively reduced TNF-α levels and regulated excessive inflammatory responses and improved pathological damage in mice with acute and chronic ulcerative colitis	Song et al.^120^
Extracellular vesicles	IL-10	mRNA	_	intravenous	delivery of IL-10 mRNA had a potent anti-inflammatory effect in IBD mice	Zhang et al.^213^

IRF8, interferon regulatory factor 8; S, size; Z, zeta potential; IBD, inflammatory bowel disease; IL, interleukin; TNF, tumor necrosis factor; MPO, myeloperoxidase; Map4k4, mitogen-activated protein kinase kinase kinase kinase 4; SNX10, sorting nexin 10; TACE, TNF-α converting enzyme.

IL-22, predominantly derived from Th17 cells, exerts a dual role in immune response enhancement (e.g., CRP and IL-8) and inhibition (e.g., antibacterial peptides and IL-10). Sugimoto et al. revealed a novel function of IL-22 in an IBD mouse model through a microinjection-based IL-22 gene-delivery approach. They found that the IL-22 gene activates STAT signaling pathways in colonic epithelial cells, leading to increased repair of goblet cells and production of mucus-related molecules. This significantly ameliorates intestinal inflammation.^118^ TNF-α, a proinflammatory cytokine mainly produced by macrophages, contributes to the etiology of IBD.^119^ Song et al. proposed a novel therapeutic option for IBD through a gene-silencing strategy using siRNAs or shRNAs to inhibit the overexpression of TNF-α, thereby reducing intestinal inflammation.^120^ Additionally, the combination of TNF-α inhibition and IL-22 enhancement has the potential to collaboratively suppress intestinal inflammation while promoting mucosal repair. Xiao et al. validated this hypothesis by the combination therapy of TNF-α siRNA (siTNF) and IL-22 gene addition in an IBD mouse model. siTNF is loaded into galactosylated polymeric nanoparticles and successfully delivered to macrophages, effectively inhibiting TNF-α expression. Meanwhile, the combination of Gal-siTNF nanoparticles and IL-22 embedded into a hydrogel demonstrates a stronger ability to suppress the expression of proinflammatory markers and encourage mucosal healing.^121^ IL-37b, an anti-inflammatory cytokine mainly secreted by macrophages or epithelial cells, possesses immunosuppressive properties that inhibit both innate and adaptive immunity.^122^ Wang et al. introduced the IL-37b gene into mesenchymal stromal cells to investigate their effectiveness in IBD mice. The results showed that IL-37b gene introduction improves the curative efficacy of mesenchymal stromal cells in IBD mice by inducing regulatory T cells (Tregs) and myeloid-derived suppressor cells, increasing IL-2 production and decreasing IFN-γ production.^123^ Moreover, IL-18 has been proven to be overexpressed in IBD and can stimulate the NF-κB signaling pathway, leading to enhanced production of proinflammatory cytokines as well as upregulation of the NOD-like receptor.^124^ An early study demonstrated that Ads expressing IL-18 antisense mRNA can dramatically reduce the activity of colitis through suppressing the production of IL-18 and IFN-α in an IBD murine model.^125^

**Figure 3:**
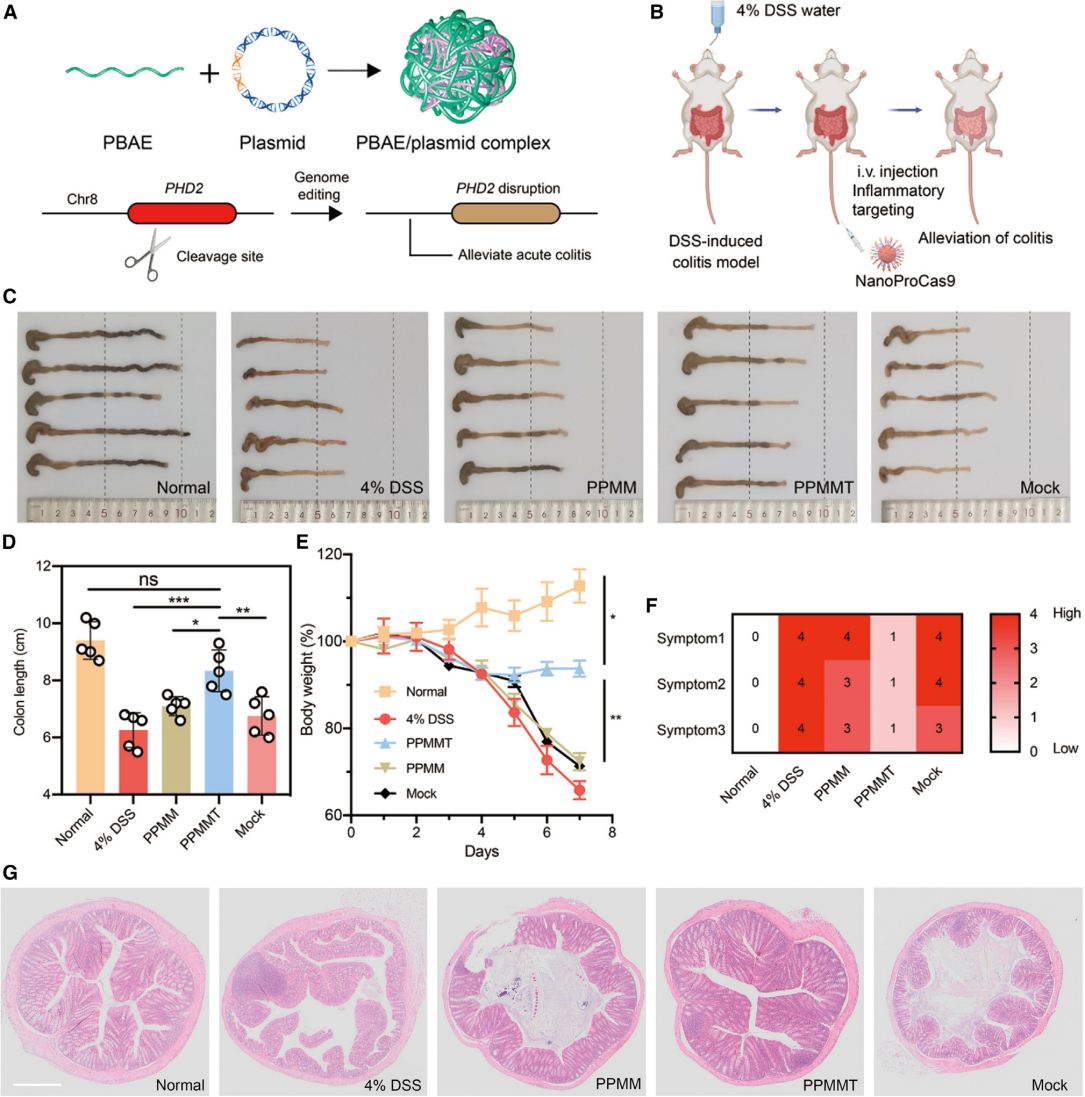
Nanomaterial delivery CRISPR-Cas9 for genome editing of PHD2 for IBD therapy (A) Cationic polymer (PBAE) in complex with Cas9 plasmid to form NanoProCas9 delivery system. (B) Dextran sodium sulfate (DSS)-induced colitis in mice injected with NanoProCas9-targeting PHD2 gene was used to assess the effect of treatment. (C) Images of the colon in each indicated treatment group; dashed lines represent per 5 cm length index. (D) Corresponding lengths after gene-editing treatment; results show that NanoProCas9-targeted PHD2 gene PPMMT restored colon length. (E) Disease activity index (DAI) of colitis in each treatment group; the PPMMT group showed higher DAI score. (F) Symptom scores for each treatment group: 1, representative weight; 2, rectal bleeding symptoms; 3, symptomatic fecal concentration symptoms. (G) Representative hematoxylin and eosin (H&E)-stained sections of colon tissues after the indicated treatment. Reprinted and modified from Yan et al.^131^ with permission. Copyright 2021, American Association for the Advancement of Science.

In addition, IFN regulatory factor 8 (IRF8) is essential for the activation of mononuclear phagocytic cells and the polarization of Th1 and Th2 cells.^126^^,^^127^ Notably, mutations in the IRF8 gene have been associated with increased susceptibility to IBD.^128^ Thus, IRF8 inhibition has been considered a potential therapy for IBD. Veiga et al. utilized antibody-targeted siRNA-loaded lipid-based nanoparticles and showed a notable decrease in IRF8 mRNA and protein levels, along with a reduction in cytokines associated with inflammation.^129^ Moreover, the roles of miRNAs in IBD are being explored, particularly in the regulation of immune responses and inflammation. Nata et al. administered miR-146b to IBD mice through intraperitoneal injection. The study revealed that overexpressing miR-146b reduces the ubiquitination of TNF receptor-associated factor proteins, leading to upregulation of NF-κB, which reduces intestinal inflammation and improves epithelial barrier function.^130^ In addition, CRISPR-Cas9-mediated modification of target genes holds great potential for the treatment of IBD. Figure 3 illustrates the targeted delivery of CRISPR-Cas9 for precision therapy of IBD by gene editing prolyl hydroxylase 2 (PHD2).^131^ Regulating the expression of disease-related genes through gene editing brings about a breakthrough in the treatment of IBD.

### Gene therapy for digestive tumors

**Figure 4:**
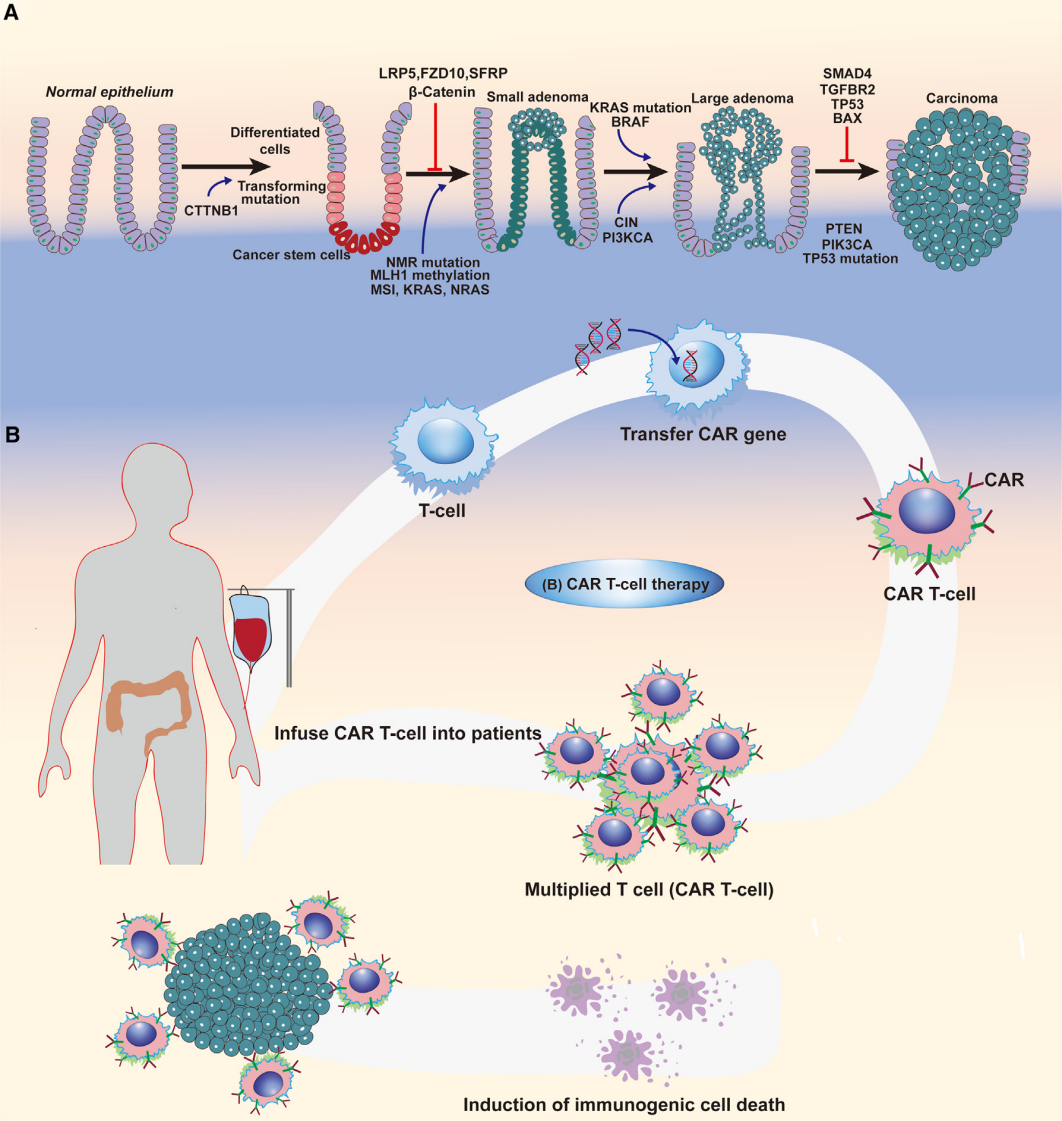
Progression process of CRC and CAR-T cell therapy for CRC (A) Proposed adenoma-to-carcinoma process in CRC and gene involvement in colorectal carcinogenesis. (B) Genetically modified CAR-T cell-directed genetic immunotherapy. T cells are removed from the patient and genetically modified in the laboratory to activate a cancer-seeking CAR receptor, then infused back into the patient. This leads to direct colorectal tumor cell death.

Digestive tumors, including esophageal, stomach, liver, hepatobiliary, pancreatic, and colorectal tumors, present significant challenges in terms of prognosis and lethality.^132^ Effective therapies are often lacking when tumor progression prevents surgical removal, highlighting the need for novel approaches to inhibit tumor progression, improve lethality, and enhance prognosis. Gene therapy represents an attractive strategy for intestinal cancers (Table 2). Various gene therapy procedures can be introduced to correct aberrant genes in tumors and halt tumor progression. These approaches include replacing tumor-suppressor genes, suppressing oncogenes, transferring suicide genes, inhibiting tumor angiogenesis, and stimulating anti-tumor immunity (Figure 4A).^133^^,^^134^

**Table 2:** Overview of delivery vectors and therapeutic targets for GI tumors

Vector	Target	Nucleic acid	Route	Result	Reference
Lipid nanoparticles	LPCAT1	siRNA	intravenous	siLPCAT1 synergistically inhibited tumor in combination with doxorubicin in a mouse model of esophageal cancer	Jun et al.^214^
Dendrimer	APC/KRAS	CRISPR-Cas9	intravenous	Cas9 ribonucleoprotein targeting APC and KRAS significantly inhibited tumor growth in CRC mouse model	Wan et al.^215^
Micelles	PCBP2	siRNA	intravenous	siPCBP2 significantly inhibited tumor progression in combination with gemcitabine in a mouse model of pancreatic cancer	Li et al.^216^
Lipidic polyplexes	IL-12	DNA	intravenous	delivery of IL12 gene effectively activated anti-cancer immune response and retarded tumor growth in an animal model of pancreatic cancer	Qiu et al.^149^
Supramolecular polymer	KRAS	Cas9	intravenous	Cas9 targeted mutant KRAS and effectively inhibited tumor growth in CRC mice	Wan et al.^217^
Chitosan nanosystem	PAK1	siRNA	intravenous	siPAK1 effectively inhibited the proliferation and metastasis of HCC cells *ex vivo* and *in vivo*	Zheng et al.^218^
Plasmid	DTA	DNA	intravenous	DTA plasmid selectively inhibited the growth of HCC cells *ex vivo* and *in vivo*	Kamimura et al.^219^
Liposome	IL-15	mRNA	intraperitoneal	delivery of IL-15 mRNA exhibited significant tumor suppressive effects in a mouse model of colon cancer	Lei et al.^195^
Magnetic nanoparticles	PD-L1	siRNA	intravenous	transfection of siPDL1 resulted in a significant reduction in tumor growth in a mouse model of pancreatic cancer	Yoo et al.^220^
Ultrasonic nanobubble	PNP	DNA	–	PNP/fludarabine suicide gene system inhibited HCC cell growth and induced apoptosis *ex vivo*	Zhang et al.^32^
Cationic liposomes	RRM2	siRNA	intravenous	siRRM2 increased sensitivity to gemcitabine treatment in a mouse model of pancreatic cancer	Zhao et al.^221^
Graphene oxide NPs	HDAC1/KRAS	siRAN	intraperitoneal	codelivery of HDAC1 and KRAS siRNA had a significant inhibitory effect on pancreatic cancer cells *ex vivo* and *in vivo*	Yin et al.^222^
Lentiviral	G6PD	shRNA	intravenous	disruption of G6PD modulated oxidation reduction and enhanced oxaliplatin-induced apoptosis in CRC cells	Ju et al.^223^
Micelles	IL-12	DNA	intratumoral	lymphocyte supernatant transfected with IL-12 inhibited CT26 cell growth *ex vivo* and significantly suppressed tumor growth in colon cancer mice	Liu et al.^224^
Polymeric NPs	VEGF	siRNA	intravenous	siVEGF significantly inhibited tumor growth in a mouse model of HCC	Wang et al.^159^
Magnetic iron oxide NPs	microRNA-21	ASO	intratumoral	ASO-miR-21 significantly induced apoptosis and inhibited the growth of pancreatic cancer cells *ex vivo* and in a mouse model of pancreatic cancer	Li et al.^225^
Adenovirus	ING4/PTEN	DNA	intratumoral	Ad-ING4/PTEN induced synergistic tumor growth inhibition and apoptosis in mouse models of gastric cancer *ex vivo* and *in vivo*	Zhang et al.^140^
Calcium phosphate nanoparticles	VEGF	siRNA	intratumoral	combination of siVEGF and fusion suicide genes exhibited potent anti-tumor activity	Liu et al.^226^
Charged polyester	KRAS	siRNA	–	the transfection of siKRAS resulted in a significant decrease in pancreatic cancer cell growth, migration, and invasion and an increase in apoptosis *ex vivo*	Yang et al.^142^
Lipid-polymer hybrid nanoparticles	HIF1α	siRNA	intravenous	codelivery of HIF1α siRNA and gemcitabine effectively inhibited the expression of HIF1α *ex vivo* and in pancreatic cancer mice, showing significant synergistic anti-tumor effects	Zhao et al.^227^
rAAV	FHL2	shRNA	intratumoral	rAAV-FHL2-shRNA showed potent anti-tumor effects in colon cancer mice, which were enhanced when combined with 5-FU treatment	Wu et al.^228^
Plasmid	GCV	HSV-TK	–	HSV-KT/GCV system significantly inhibited the growth of HCC cells *ex vivo*	Qu et al.^145^
Lentivirus	STAT3	shRNA	intraperitoneal	STAT3 silencing enhanced the efficacy of suicide gene therapy in CT26 cell xenograft mice	Ahn et al.^148^

LPCAT1, lysophosphatidylcholine acyltransferase 1; CRC, colorectal cancer; PCBP2, poly(RC) binding protein 2; IL, interleukin; PAK1, p21 protein-activated kinase 1; DTA, diphtheria toxin fragment A; HCC, hepatocellular carcinoma; PD-L1, programmed death-ligand 1; PNP, purine nucleoside phosphorylase; RRM2, ribonucleotide reductase subunit 2; HDAC1, histone deacetylase 1; G6PD, glucose-6-phosphate dehydrogenase; VEGF, vascular endothelial growth factor; ASO, antisense oligonucleotides; ING4, inhibitor of growth 4; PTEN, phosphatase and tension homolog gene; HIF1α, hypoxia-inducible factor 1α; FHL2, four and a half LIM-only protein 2; GCV, ganciclovir; HSV-TK, herpes simplex virus thymidine kinase; STAT3, signal transducer and activator of transcription 3.

The most common genetic alterations in digestive tumors involve mutations in the p53 gene. Inhibiting the function of mutant p53 is considered a potent approach to impede malignant tumor progression. Cui et al. have explored the therapeutic effects of combining gene therapy with p53 and conventional treatments (such as chemotherapy) in patients with middle- to advanced-stage esophageal cancer. Ad-p53 vector is administered endoscopically into the malignancy. The findings demonstrate that combination therapy considerably reduces the tumor volume compared with chemotherapy alone, with slight side effects.^135^ Another tumor-suppressor gene with phosphatase activity, PTEN, plays a crucial role in cell proliferation, adhesion, migration, angiogenesis, and apoptosis.^136^ Loss of PTEN expression leads to a deficient phosphatase activity, encouraging oncogenesis and tumor progression in digestive tumors.^137^^,^^138^ For example, Xiao et al. conducted research on modified Ad5-PTEN by the epithelial cell adhesion molecule (EpCAM) aptamer EpDT3 to treat HCC (EpCAM is a surface marker of cancer stem cells in HCC). The study demonstrates that PTEN significantly inhibits the proliferation and migration of HepG2 HCC cells and exhibits potent anti-tumor activity in an aggressive HepG2 xenograft mouse model.^139^ Furthermore, the combination of PTEN and the tumor-suppressor gene inhibitor of growth 4 (ING4) has been shown to be therapeutically effective in stomach cancer. Zhang et al. constructed a recombinant Ad coexpressing ING4 as well as PTEN (AdVING4/PTEN) for the treatment of gastric carcinoma. They discovered that AdVING4/PTEN synergistically suppresses tumors by upregulating tumor-suppressing genes (e.g., p53, p21, Bax, etc.) and downregulating VEGF, thereby inhibiting angiogenesis. Therefore, combining ING4 and PTEN for gene therapy may represent an effective approach for treating human gastric carcinoma and others related tumors.^140^

The K-Ras oncogene is highly prevalent in pancreatic cancer, present in approximately 95% of cases. Mutations in K-Ras result in increased proliferation and resistance to apoptosis, occurring early in pancreatic cell transformation and tumor progression.^29^ Targeting K-Ras mutations in pancreatic cancer through siRNA-mediated gene silencing represents a potential therapeutic approach. However, naked siRNAs are negatively charged and prone to nuclease-induced degradation, necessitating suitable carriers for efficient delivery to target cells.^141^ In 2015, Yang et al. proposed a biodegradable and charged polyester-based vehicle that facilitates the transfer of K-Ras siRNA into pancreatic carcinoma cells. These biodegradable and biocompatible vectors successfully transduce mutant K-Ras-targeting siRNA into MiaPaCa-2 cells with high efficiency. This leads to the regulation of K-Ras downstream genes, notably weakening the growth, migration, and invasion abilities of pancreatic cancer cells while promoting apoptosis.^142^ Suicide gene therapy is another strategy employed in gene therapy, particularly for malignancies that are unresponsive to conventional treatments, such as pancreatic cancer and HCC.^143^^,^^144^^,^^145^ For instance, magnetic MSNPs have been utilized for HSV-TK/GCV suicide gene delivery in HCC treatment, enhancing the therapeutic efficacy of suicide gene therapy.^146^ Hiraoka et al. injected replication-competent retrovirus vectors carrying the yeast cell nucleotide deaminase gene into a multifocal CRC metastasis model. The gene converts the harmless prodrug 5-FC into the chemotherapeutic agent 5-FU, resulting in significant inhibition of tumor growth.^147^ Silencing STAT3 not only suppresses tumor cell proliferation and promotes anti-tumor immune responses but also enhances the anti-cancer efficacy of tumor suicide gene therapy.^148^

However, the therapy effects based on tumor-suppressor genes, oncogenes, and suicide genes have limitations and can elicit immunogenicity. In such cases, there is high anticipation for enhancing anti-tumor immunotherapy, which includes cytokines, CAR-T cells, tumor vaccines, and more.^149^^,^^150^^,^^151^ CAR-T cell therapy brings fresh impetus to the field of anti-tumor immunotherapy. T cells are genetically modified to express synthetic receptors that enhance their ability to target and kill cancer cells, ultimately leading to tumor destruction (Figure 4B).^150^ Notably, T cells for CAR-T cell therapy can be derived from autologous or allogeneic sources. Autologous CAR-T cells have shown exciting clinical results in the field of GI tumor immunotherapy due to their stability, low immunogenicity, and persistence.^152^ A case report has demonstrated significant regression of visceral metastases in a patient with advanced metastatic pancreatic carcinoma who has received an infusion of genetically modified self T cells specifically targeting the tumor-expressed mutation KRASG12D through a clonal expression of two heteromeric HLA-C∗08:02-restricted T cell receptors. The response lasts for up to 6 months.^153^ Nevertheless, autologous CAR-T cell therapy still faces some unavoidable issues, including high cost, long manufacturing cycles, and restricted cell sources.^154^ Consequently, allogeneic CAR-T cell therapy has emerged as a potential alternative. Graft-versus-host disease (GvHD) is one of the main issues in the allogeneic approach. CRISPR-Cas9 gene-editing technologies are being employed to address this challenge, such as the knockout of T cell receptor (TCR).^154^ This advancement has significantly broadened the applicability of gene therapy in oncology treatment through allogeneic CAR-T cell therapy based on CRISPR-Cas9 technology, which has exhibited remarkable efficacy in hematologic tumors. However, CAR-T cells still face some hurdles in the solid tumor microenvironment, such as target antigen heterogeneity, physical and metabolic barriers, tumor-derived soluble factors and cytokines, immunosuppressive cells, and more.^155^ Novel approaches are being explored to overcome these challenges, such as multifunctional CARs, antigen-specific CARs, and inhibiting Tregs.^156^^,^^157^

Tumors are highly vascularized, and angiogenesis plays a critical role in tumor development, progression, and metastasis. In light of this, anti-tumor angiogenesis gene therapy has emerged as an attractive approach for tumor suppression. Various growth factors, with VEGF being a dominant player, have been implicated in tumor angiogenesis.^158^ VEGF siRNA (siVEGF) has shown promise as a candidate for anti-angiogenic therapy in tumors. Wang et al. conducted a study using a biodegradable cationic polymer-mediated delivery system for siVEGF in an HepG2 tumor xenograft model. They observed a substantial reduction in VEGF expression at both the mRNA and protein levels, resulting in inhibited tumor growth.^159^ While these gene therapy strategies have demonstrated effectiveness in suppressing tumors in animal models, their safety and efficacy in humans still require further validation. This validation process depends on factors such as the therapeutic gene, vector type, dose, administration route, and tumor type. Tumorigenesis is a complex, multifactorial process, making it challenging to identify a single target gene. Combination therapies, which involve the use of multiple treatment modalities, may prove more effective than gene therapy alone in addressing the complexity of tumorigenesis.

Oncolytic virotherapy is a burgeoning approach within cancer immunotherapy, which is also considered a specific gene therapy strategy. The approach employs modified viruses to selectively infect and eradicate tumor cells, yet it rarely affects healthy cells. Additionally, oncolytic virotherapy can elicit anti-tumor immune responses by releasing tumor-associated antigens (TAAs) and activating systemic inflammatory reactions.^160^ Several viruses, such as Ads, herpesvirus, and vaccinia virus, have been developed as oncolytic viruses.^161^ Genetic engineering techniques have been employed to improve the targeting specificity and safety of these viruses. Typical genetic modification strategies encompass gene deletion or insertion, targeted modification, and safety enhancement.^160^ Pexa-Vec (JX-594) is a genetically engineered vaccinia virus that selectively replicates in tumor cells by deleting the TK gene, thereby reducing adverse effects on healthy cells. Moreover, the insertion of the granulocyte-macrophage colony-stimulating factor (GM-CSF) gene into the viral genome enables Pexa-Vec to induce GM-CSF release after infecting tumor cells, thereby activating the host immune system against tumors. Results from a phase 1 clinical trial of Pexa-Vec in HCC demonstrate positive effects.^162^ Subsequently, a randomized phase 2 clinical trial was conducted to explore the optimal dose of Pexa-Vec for the treatment of HCC. The intrahepatic response rates are comparable between the high- and low-dose groups (62%), with median survival of 14.1 and 6.7 months, respectively. These results underscore the great anti-tumor activity of Pexa-Vec in HCC.^163^ Another example is VCN-01, a genetically engineered type 5 oncolytic Ad that replicates and expresses the hyaluronidase PH20 in cancer cells with a dysfunctional RB1 pathway. In patients with pancreatic cancer, VCN-01 combined with chemotherapy shows improved anti-tumor effects and disease stabilization.^164^ Similarly, a genetically modified oncolytic Ad, H101, shows promising results when combined with anti-PD-1 antibodies in a mouse model of CRC, leading to reduced tumor volume, increased infiltration of CD8^+^ T cells, and enhanced anti-tumor immune response.^165^ Despite the promising clinical outcomes of genetically engineered oncolytic viruses, several challenges persist, such as host antiviral immune responses, limited tumor penetration, short persistence, safety concerns, and tumor heterogeneity. These obstacles hinder the clinical translation of oncolytic virotherapy.^160^ Fortunately, genetically engineered multifunctional oncolytic viruses and combination therapeutic strategies, such as combining chemotherapy, immune checkpoint inhibitors, or CAR-T therapy, hold promise in overcoming these challenges and advancing precise anti-tumor therapy.^166^

### Gene therapy for other GI diseases

Transfer of therapeutic genes into hepatocytes presents a promising and innovative approach for treating liver diseases, such as viral hepatitis, liver fibrosis, and cirrhosis. Chronic viral hepatitis, particularly hepatitis B and C, poses a significant global public health challenge, increasing the risk of cirrhosis and HCC. Considerable improvements have been achieved with antiviral drugs. For example, pegylated interferon-α (INF-α) administration results in sustained INF-α levels in the bloodstream with a single weekly injection.^167^ Moreover, nucleoside and nucleotide analogs efficiently inhibit the replication of hepatitis B.^168^ Antiviral drugs, while beneficial, face limitations due to the emergence of drug resistance and associated side effects, impeding the achievement of complete viral clearance. Given this existing therapeutic conundrum, gene therapy emerges as an effective and well-tolerated approach for treating chronic hepatitis B and C. The persistent presence of cccDNA within the nuclei of infected cells drives the progression of HBV-related illnesses.^169^ Hence, targeting and disabling cccDNA through gene-editing technologies is a viable strategy for HBV cure. Lin et al. explored the CRISPR-Cas9 system, employing eight synthetic guide RNAs (gRNAs) designed to target HBV genomes of genotype A. Remarkably, the CRISPR-Cas9 system drastically decreases the generation of the HBV genome in the HBV persistence mouse model.^170^ Differing from HBV, hepatitis C virus (HCV) is an RNA virus that exclusively reproduces in the cytoplasm of infected cells without integrating into the host genome. RNA interference (RNAi)-based gene silencing and anti-miRNA oligonucleotides (such as miRNA-122) have shown therapeutic potential against HCV.^171^^,^^172^ Mounting evidence has demonstrated that siRNAs can significantly reduce the expression levels of target genes and highly inhibit HCV replication both *ex vivo* and *in vivo*.^173^^,^^174^^,^^175^ Additionally, miRNA-122, which is specifically expressed in hepatocytes, has been found to be closely associated with HCV replication.^176^ Accordingly, anti-miRNA oligonucleotides (anti-miRs) broaden prospects for HCV therapy. However, issues such as targeting and stability hinder the *in vivo* application of anti-miRs. To overcome these challenges, Fu et al. designed monomethoxy (polyethylene glycol)-poly (d,l lactide-co-glycolide)-poly (l-lysine) (mPEG-b-PLGA-b-PLL) nanoparticles for the delivery of miR-122 antagomir. This nanoparticle-based system effectively reduces miR-122 expression, enabling RNAi therapy for HCV.^177^

Moreover, metabolic liver diseases, characterized by genetic abnormalities in the liver, pose a significant burden, particularly in children, with limited treatment options. Although liver transplantation has achieved excellent results, invasive procedures are closely linked with high morbidity and mortality. It is essential to search for alternative treatments that are both less invasive and more effective, such as gene therapy. PFIC3, caused by gene defects in the ABCB4 gene, represents a suitable target for gene therapy. Data from several studies point to excellent results for gene therapy in PFIC3.^60^^,^^178^ Aronson et al. used AAV8-mediated hABCB4 gene expression to restore phospholipid transport and improve cholestasis and liver damage in a mouse model of PFIC3. The introduction of AAV8-hABCB4 reduces hepatocyte proliferation and prevents the progression of liver fibrosis.^179^

Peptic ulcers, which are inflammatory defects of the GI mucosa, can lead to complications such as bleeding and perforation. In addition to treating the underlying causes, promoting ulcer healing is a key focus. This includes the regeneration and repair of epithelial cells and angiogenesis. Angiopoietin-1 (Ang-1) and VEGF play crucial roles in angiogenesis by facilitating the delivery of oxygen and nutrients to healing areas. Stimulation of angiogenesis can accelerate peptic ulcer healing. In a gastric ulcer rat model, Jones et al. introduced non-viral DNA expressing VEGF and/or Ang-1 into ulcer sites, resulting in increased angiogenesis and accelerated ulcer healing.^180^ Similarly, in a rat model of chronic duodenal ulcer, administration of adenoviral vectors encoding VEGF genes promotes ulcer healing without affecting stomach acid secretion.^181^

Acute pancreatitis arises from pancreatic damage induced by early trypsinogen activation due to a variety of reasons, resulting in self-digestion of pancreatic tissue, with escalating morbidity and mortality.^182^ Accumulating evidence suggests that proinflammatory factors, such as IL-1β, IL-6, TNF-α, and IL-18, contribute to the development of pancreatitis.^183^^,^^184^ Notably, IL-10 serves as an anti-inflammatory cytokine that inhibits the production and activity of proinflammatory cytokines. A pioneering study demonstrated that human IL-10 gene therapy significantly reduces the severity and mortality of pancreatitis in rats.^185^ ER stress is another major pathogenetic component in acute pancreatitis. Previous studies have shown that the activation of the recombinant activating transcription factor 6 (ATF6) gene is involved in ER stress-induced apoptosis and injury of acinar cells by regulating the p53/AIFM2 pathway. Thus, ATF6 siRNA emerges as a promising therapeutic option for severe acute pancreatitis.^186^ Furthermore, the abdominal pain caused by chronic pancreatitis is excruciating and can even drive individuals to contemplate suicide. Although opioids remain the primary non-surgical treatment for pancreatic pain, their use is limited due to side effects such as addiction, constipation, nausea, and tolerance. Gene therapy offers a novel alternative for pancreatitis pain management. One potential gene therapy approach involves the utilization of HSV-1-based viral vectors to deliver the met-enkephalin gene directly to the patient’s abdominal ganglia via endoscopy or the gastric wall. This method results in increased expression of enkephalin and the alleviation of chronic pancreatitis pain, proving a definite hope for effectively managing pain in clinical pancreatitis.^187^

## Clinical implementation of gene therapy

In 1990, William French Anderson led the initial clinical study of gene therapy for severe combined immunodeficiency illnesses.^10^ Subsequent clinical trials have been conducted on gene therapy for digestive disorders, as outlined in Table 3. One notable gene therapy approach is Rexin-G, a chimeric retroviral vector that expresses a dominant-negative cell-cycle protein G1 gene to specifically target and destroy solid tumors. In 2004, the first clinical trial of Rexin-G as a gene drug was conducted, involving three patients with stage IV pancreatic cancer. The trial was intended to assess the safety and anti-tumor efficacy of Rexin-G. The study demonstrated tumor growth inhibition in all three patients, with no observed dose-limiting toxicity. Despite the small sample size, the results were encouraging.^188^ Subsequently, a phase 1/2 clinical investigation (ClinicalTrials.gov: NCT00504998) was conducted to determine the safety and optimal dosage of Rexin-G for recurrent or advanced pancreatic cancer that is unresponsive to gemcitabine. CAR-T cell immunotherapy, which involves equipping T cells with tumor CARs to precisely target and eliminate tumor cells, has also been explored in human studies. Clinical development of CAR-T cell therapy for GI tumors, including gastric, colorectal, liver, and pancreatic cancers, has been slow due to the greater heterogeneity of solid tumor antigens and the immunosuppressive tumor microenvironment.^189^ In the first human trial of CAR-T cells for solid tumors conducted in the 1990s, CAR-T72 cells were engineered to recognize tumor-associated glycoprotein (TAG)-72, a biomarker frequently expressed in solid tumors, which were administered directly through the hepatic artery. This was a phase 1 trial involving 14 patients with CRC liver metastases that found that CAR-T72 cells exhibit short persistence (less than 14 weeks) after administration and show a tendency to migrate to tumor tissue. Despite immunogenicity emerges, it is associated with rapid clearance of subsequent CAR-T72 cell infusion. Therefore, the study demonstrated the relative safety of CAR-T72 cells.^190^ Recently, a phase 1 clinical trial (ClinicalTrials.gov: NCT03874897) got underway to evaluate the safety of CLDN18.2-targeted CAR-T cells (CT041) in patients with GI tumors. Interim results have shown an overall response rate (ORR) of approximately 50%, with higher response rates observed in gastric cancer. Hematologic toxicity of grade 3 or higher is observed in all patients, and most experience low-grade cytokine release syndrome.^191^ Although this toxic effect is acceptable, larger clinical studies are still needed to thoroughly investigate the safety and efficacy of CAR-T in GI tumors. Moreover, IL-10, a factor that reduces inflammation, is crucial in the treatment and prevention of IBD. An early clinical trial has shown that local liposome-mediated IL-10 DNA transfer effectively inhibits the production of proinflammatory factors in patients with severe IBD and increases IL-10 concentrations over an extended period, thus avoiding toxic systemic side effects by local administration.^192^ To further enhance safety, a phase 1 clinical trial using IL-10-expressing transgenic bacteria (LL-Thy12) to treat patients with Crohn’s disease has been conducted to evaluate its safety and efficacy. The results showed only mild adverse effects of LL-Thy12 therapy and a reduction in disease activity, holding promise for future maintenance treatment of gene therapy for IBD.^193^ Although numerous animal models have shown the great potential of gene therapy, its safety and efficacy in humans are highly ambiguous, particularly considering racial differences. Therefore, future studies should focus on conducting more extensive and larger clinical trials to verify the efficacy and safety of gene therapy in human populations.

**Table 3:** Summary of clinical trials for GI disease gene therapy

Disease	Vector/strategy	Delivery route	Phase (patient)	Study status/result	Trial code/ref.
Pancreatic cancer	adenovirus/HSV-TKarm A: HSK −TK + valacyclovirarm B: HSV−TK + valacyclovir + chemoradiation	intratumoral	1 (24)	completed/well tolerated; median OS: 10 months in arm A and 12 months in arm B with 25% of RECIST response	NCT00638612 Aguilar et al.^229^
	plasmid/CYL-02 + gemcitabine	intratumoral	1 (22)	completed/well tolerated; 12 SD; OS in non-metastatic patients: 12.6 months	NCT01274455 Buscail et al.^230^
	adenovirus/cancer vaccine/GVAX + CRS 207 (arm A) vs. GVAX alone (arm B)	subcutaneous	2 (90)	completed/local reactions 77%; general minor AE: 53%–62%; OS: 6.1 months in arm A vs. 3.9 months in arm B	NCT01417000 Le et al.^231^
	reovirus/Reolysin + paclitaxel + carboplatin (arm A) vs. paclitaxel + carboplatin (arm B)	intravenous	2 (73)	completed/well tolerated; no difference in PFS and OS between the two arms	NCT01280058 Noonan et al.^232^
	adenovirus/TherageneAd5-yCD/mutTKSR39rep-ADP	intratumoral	1 (9)	completed/well tolerated; median PFS: 11.4 months	NCT02894944 Lee et al.^233^
	adenovirus/TherageneAd5-yCD/mutTKSR39rep-ADP + radiation	–	2 (12)	recruiting	NCT04739046
	plasmid/SGT-53 + nab-paclitaxel + gemcitabine	intravenous	2 (28)	recruiting	NCT02340117
	adenovirus/LOAd703 + chemotherapy (arm 1) vs. LOAd703 + chemotherapy + atezolizumab (arm2)	intratumoral	1/2a (55)	recruiting	NCT02705196
HCC	adenovirus/HSV-TK	intratumoral	1 (10)	completed/well tolerated and safe; 100% feasible; no PR; 60% SD	NCT00844623 Sangro et al.^234^
	JX-594(Pexa-Vec): recombinant vaccinia virus	intratumoral	2 (30)	completed/RECIST: 15%; median survival of 14.1 months compared with 6.7 months on the high and low doses, respectively; hazard ratio: 0.39; p = 0.020	NCT00554372 Heo et al.^163^
	experimental: JX-594(Pexa-Vec): recombinant vaccinia virus + sorafenibactive comparator: sorafenib	intratumoral	3 (459)	completed/no results published	NCT02562755
	GLYCAR T cells + fludarabine and Cytoxan	intravenous	1 (9)	completed/no results published	NCT02905188
	GPC3- and/or TGF-β-targeting CAR-T cells	intravenous	1 (30)	recruiting	NCT03198546
	GPC3-targeted CAR-T cells	intravenous	1 (38)	recruiting	NCT05003895
CRC	adenovirus/hIFN-β	intravenous	1/2 (44)	completed/no results published	NCT0010786.1
Esophageal cancer	CRISPR-Cas9/PD-1 knockout T cells	intravenous	– (16)	completed/no results published	NCT03081715
	TNFerade + 5-FU + radiation	intravenous	2 (24)	completed/well tolerated; median OS: 47.8 months; the 3- and 5-year OS rates and DFS rates were 54% and 41% and 38% and 38%, respectively	NCT00051480 Chang et al.^235^
Stomach cancer	adenovirus/DC-CEA-specific cytotoxic T lymphocytes	intravenous	1 (60)	active, not recruiting	NCT02496273
	KK-LC-1 TCR-T cells	intravenous	1 (42)	recruiting	NCT05483491
GI tumors	CAR-CLDN18.2 T cells + PD-1 monoclonal antibody + chemotherapy	intravenous	1 (123)	recruiting/interim results: well tolerated and safe; ORR and DCR reached 48.6% and 73.0%, respectively; the 6-month duration of response rate was 44.8%	NCT03874897 Qi et al.^191^
Very-early-onset IBD	cord blood stem cell transplantation	intravenous	1 (50)	active, not recruiting/interim results: 9 patients received transplantation; complete remission: 67%	NCT04170192 Peng et al.^236^

HSV-TK, herpes simplex virus thymidine kinase; OS, overall survival; RECIST, response evaluation criteria in solid tumors; SD, stable disease; AE, adverse event; PFS, progression-free-survival; HCC, hepatocellular carcinoma; PR, partial response; GPC3, Glypican-3; TGF, transforming growth factor; CAR-T, chimeric antigen receptor T cell; CRC, colorectal cancer; INF, interferon; TNF, tumor necrosis factor; DFS, disease-free-survival; GI, gastrointestinal; ORR, overall response rate; DCR, disease control rate; IBD, inflammatory bowel disease.

## Conclusion and prospects

Gene therapy, a groundbreaking treatment approach, involves introducing specific genes into targeted cells to repair and enhance malfunctioning genes. Accumulating data illustrate that gene therapy is gaining momentum in GI diseases, and a comprehensive understanding of the underlying principles and techniques of gene therapy can offer novel insights into the treatment of digestive disorders. In this review, firstly, we provide a comprehensive overview of the gene therapy targets and signaling pathways for GI diseases, as the accurate selection of targets is crucial for the efficacy of gene therapy. Besides, we introduce the vectors used for gene delivery, including AAVs, nanoparticles, and hydrogel, and highlight the advantages and limitations as well as *in vivo* and *ex vivo* challenges associated with these vectors. Finally, we summarize the application of gene therapy in digestive diseases, involving various gene therapy strategies. Overall, gene therapy holds immense promise as a therapeutic option for digestive diseases, particularly advanced GI tumors and IBD.

Preliminary studies have provided evidence demonstrating the safety and effectiveness of gene therapy for GI disorders. Indeed, gene therapy has the potential to profoundly change the prognosis of debilitating and often fatal diseases by offering durable and curative solutions. For instance, silencing the synaptotagmin XIII (SYT13) gene using siRNA significantly reduces the invasive and migratory capabilities of gastric cancer cells.^194^ Moreover, liposome-based delivery of an IL-15 mRNA vector successfully stimulates lymphocytes and results in significant inhibition of CRC.^195^ Importantly, these factors need to be taken into account to ensure the success (e.g., safety, durability, and efficacy) of gene therapy for GI diseases, including target gene selection, duration of gene expression, vector tolerance and immunogenicity, and gene-delivery methods. The selection of the optimal target gene is critical for the success of gene therapy, considering the diverse pathogenesis of digestive diseases. As our understanding of the pathophysiology of these diseases continues to grow, more effective target genes for gene therapy will be identified. Prolonging the duration of transgene expression is also crucial to extend the treatment cycle and reduce the need for repeated administrations. AAV vectors offer the longest duration of expression, while non-viral vectors generally provide relatively shorter durations. In addition, stem cell transfection is an effective method to extend expression time. The tolerability and immunogenicity of vectors should be taken into account during the selection process. Non-viral vectors have no loading capacity limitation and are rapidly evolving to increase cell transfection rates and maximize therapeutic efficacy while minimizing toxic side effects. The gene-delivery method is another important factor to consider. The inherent properties of the intestine make it an attractive candidate for therapeutic gene transfer. The intestine possesses a large surface area; is easily accessible through oral, rectal, or endoscopic administration; contains stem cells in the crypts; and has a highly vascularized gut epithelium.^196^ Therefore, the development of new technologies, vectors, and gene targets significantly advances the treatment of GI diseases.

Before advancing gene therapies into clinical trials, it is crucial to thoroughly evaluate potential risks and issues. One significant risk associated with integrating vectors is the possibility of insertional mutagenesis. While AAV vectors have a low risk of insertional mutations, they can trigger hepatic oncogenic gene overexpression through multiple mechanisms.^197^ To mitigate this risk, the development of safer vectors, such as non-viral vectors, can be pursued. Additionally, AAV vectors have limitations, including the inability to sustain replication and the constraint on the length of encapsulated genes (less than 5 kb). In the case of vectors administered *in vivo*, pre-existing antibodies and delayed cellular immune responses can lead to the destruction of target cells and failure of therapeutic efficacy. Risk of immune response can be reduced by pre-exclusion of antibodies and adjuvant immunomodulatory drugs *in vivo*. Furthermore, the cost of production is regarded as a real issue that may stymie the development of gene therapy. Therefore, there is great concern about the lasting advantages associated with these high-priced once-only gene therapies.

Taken together, gene therapy holds tremendous promise for the treatment of currently incurable digestive diseases. However, despite significant progress in preclinical studies, several challenges need to be addressed before gene therapy can be translated into clinical practice. These challenges include cost effectiveness, long-term safety, and immune responses. As gene-editing and -delivery technologies continue to advance, future efforts should focus on the development of more efficient vectors and the identification of new therapeutic targets to improve the clinical translation success of gene therapy.

## References

[1] Gough N.R., Xiang X., Mishra L. (2021). TGF-β Signaling in Liver, Pancreas, and Gastrointestinal Diseases and Cancer. Gastroenterology.

[2] Long F., Lin Z., Li L., Ma M., Lu Z., Jing L., Li X., Lin C. (2021). Comprehensive landscape and future perspectives of circular RNAs in colorectal cancer. Mol. Cancer.

[3] McGovern D.P.B., Kugathasan S., Cho J.H. (2015). Genetics of Inflammatory Bowel Diseases. Gastroenterology.

[4] Liebe R., Esposito I., Bock H.H., Vom Dahl S., Stindt J., Baumann U., Luedde T., Keitel V. (2021). Diagnosis and management of secondary causes of steatohepatitis. J. Hepatol..

[5] Camilleri M., Zhernakova A., Bozzarelli I., D’Amato M. (2022). Genetics of irritable bowel syndrome: shifting gear via biobank-scale studies. Nat. Rev. Gastroenterol. Hepatol..

[6] High K.A., Roncarolo M.G. (2019). Gene Therapy. N. Engl. J. Med..

[7] Pickar-Oliver A., Gersbach C.A. (2019). The next generation of CRISPR-Cas technologies and applications. Nat. Rev. Mol. Cell Biol..

[8] Bulcha J.T., Wang Y., Ma H., Tai P.W.L., Gao G. (2021). Viral vector platforms within the gene therapy landscape. Signal. Transduct. Target. Ther..

[9] Ramamoorth M., Narvekar A. (2015). Non viral vectors in gene therapy- an overview. J. Clin. Diagn. Res..

[10] The ADA human gene therapy clinical protocol (1990). Hum. Gene Ther. 1, 327–362. 10.1089/hum.1990.1.3-327. 2081198

[11] Payen E. (2022). Efficacy and Safety of Gene Therapy for β-Thalassemia. N. Engl. J. Med..

[12] Tebas P., Stein D., Tang W.W., Frank I., Wang S.Q., Lee G., Spratt S.K., Surosky R.T., Giedlin M.A., Nichol G. (2014). Gene editing of CCR5 in autologous CD4 T cells of persons infected with HIV. N. Engl. J. Med..

[13] Mason D., Chen Y.-Z., Krishnan H.V., Sant S. (2015). Cardiac gene therapy: Recent advances and future directions. J. Control Release..

[14] O’Connor D.M., Boulis N.M. (2015). Gene therapy for neurodegenerative diseases. Trends Mol. Med..

[15] Camilleri A.E., Nag S., Russo A.R., Stiles K.M., Crystal R.G., Pagovich O.E. (2021). Gene therapy for a murine model of eosinophilic esophagitis. Allergy.

[16] Kastenhuber E.R., Lowe S.W. (2017). Putting p53 in Context. Cell.

[17] Liebl M.C., Hofmann T.G. (2021). The Role of p53 Signaling in Colorectal Cancer. Cancers.

[18] Nakayama M., Oshima M. (2019). Mutant p53 in colon cancer. J. Mol. Cell Biol..

[19] Senzer N., Nemunaitis J., Nemunaitis D., Bedell C., Edelman G., Barve M., Nunan R., Pirollo K.F., Rait A., Chang E.H. (2013). Phase I study of a systemically delivered p53 nanoparticle in advanced solid tumors. Mol. Ther..

[20] Weintraub S.J., Prater C.A., Dean D.C. (1992). Retinoblastoma protein switches the E2F site from positive to negative element. Nature.

[21] Narasimha A.M., Kaulich M., Shapiro G.S., Choi Y.J., Sicinski P., Dowdy S.F. (2014). Cyclin D activates the Rb tumor suppressor by mono-phosphorylation. eLife.

[22] Serra S., Chetty R. (2018). p16. J. Clin. Pathol..

[23] Caldas C., Hahn S.A., da Costa L.T., Redston M.S., Schutte M., Seymour A.B., Weinstein C.L., Hruban R.H., Yeo C.J., Kern S.E. (1994). Frequent somatic mutations and homozygous deletions of the p16 (MTS1) gene in pancreatic adenocarcinoma. Nat. Genet..

[24] Sherr C.J., Roberts J.M. (1999). CDK inhibitors: positive and negative regulators of G1-phase progression. Genes Dev..

[25] Yang Z., Hu J., Li D., Pan X. (2016). Adenovirus with p16 gene exerts antitumor effect on laryngeal carcinoma Hep2 cells. Mol. Med. Rep..

[26] Zhao S., Mi Y., Guan B., Zheng B., Wei P., Gu Y., Zhang Z., Cai S., Xu Y., Li X. (2020). Tumor-derived exosomal miR-934 induces macrophage M2 polarization to promote liver metastasis of colorectal cancer. J. Hematol. Oncol..

[27] Leng A., Liu T., He Y., Li Q., Zhang G. (2009). Smad4/Smad7 balance: a role of tumorigenesis in gastric cancer. Exp. Mol. Pathol..

[28] Murugan A.K., Grieco M., Tsuchida N. (2019). RAS mutations in human cancers: Roles in precision medicine. Semin. Cancer Biol..

[29] Lisiansky V., Naumov I., Shapira S., Kazanov D., Starr A., Arber N., Kraus S. (2012). Gene therapy of pancreatic cancer targeting the K-Ras oncogene. Cancer Gene Ther..

[30] Lanfredini S., Thapa A., O’Neill E. (2019). RAS in pancreatic cancer. Biochem. Soc. Trans..

[31] Wildner O., Blaese R.M., Morris J.C. (1999). Therapy of colon cancer with oncolytic adenovirus is enhanced by the addition of herpes simplex virus-thymidine kinase. Cancer Res..

[32] Zhang B., Chen M., Zhang Y., Chen W., Zhang L., Chen L. (2018). An ultrasonic nanobubble-mediated PNP/fludarabine suicide gene system: A new approach for the treatment of hepatocellular carcinoma. PLoS One.

[33] Zhou J.h., Tang B., Liu X.l., He D.w., Yang D.t. (2007). hTERT-targeted E. coli purine nucleoside phosphorylase gene/6-methylpurine deoxyribose therapy for pancreatic cancer. Chin. Med. J..

[34] Klopp A., Schreiber S., Kosinska A.D., Pulé M., Protzer U., Wisskirchen K. (2021). Depletion of T cells via Inducible Caspase 9 Increases Safety of Adoptive T-Cell Therapy Against Chronic Hepatitis B. Front. Immunol..

[35] Sun W., Li Z., Zhou X., Yang G., Yuan L., Wang Y., Qiao L. (2019). Advances in the techniques and methodologies of cancer gene therapy. Drug Deliv..

[36] Ferrara N., Adamis A.P. (2016). Ten years of anti-vascular endothelial growth factor therapy. Nat. Rev. Drug Discov..

[37] Apte R.S., Chen D.S., Ferrara N. (2019). VEGF in Signaling and Disease: Beyond Discovery and Development. Cell.

[38] Zhong M., Li N., Qiu X., Ye Y., Chen H., Hua J., Yin P., Zhuang G. (2020). TIPE regulates VEGFR2 expression and promotes angiogenesis in colorectal cancer. Int. J. Biol. Sci..

[39] Han L., Lin X., Yan Q., Gu C., Li M., Pan L., Meng Y., Zhao X., Liu S., Li A. (2022). PBLD inhibits angiogenesis via impeding VEGF/VEGFR2-mediated microenvironmental cross-talk between HCC cells and endothelial cells. Oncogene.

[40] Tarnawski A.S., Ahluwalia A. (2021). The Critical Role of Growth Factors in Gastric Ulcer Healing: The Cellular and Molecular Mechanisms and Potential Clinical Implications. Cells.

[41] Vignali D.A.A., Kuchroo V.K. (2012). IL-12 family cytokines: immunological playmakers. Nat. Immunol..

[42] Kobayashi M., Fitz L., Ryan M., Hewick R.M., Clark S.C., Chan S., Loudon R., Sherman F., Perussia B., Trinchieri G. (1989). Identification and purification of natural killer cell stimulatory factor (NKSF), a cytokine with multiple biologic effects on human lymphocytes. J. Exp. Med..

[43] Trinchieri G. (1994). Interleukin-12: a cytokine produced by antigen-presenting cells with immunoregulatory functions in the generation of T-helper cells type 1 and cytotoxic lymphocytes. Blood.

[44] Tugues S., Burkhard S.H., Ohs I., Vrohlings M., Nussbaum K., Vom Berg J., Kulig P., Becher B. (2015). New insights into IL-12-mediated tumor suppression. Cell Death Differ..

[45] Aparicio C., Belver M., Enríquez L., Espeso F., Núñez L., Sánchez A., de la Fuente M.Á., González-Vallinas M. (2021). Cell Therapy for Colorectal Cancer: The Promise of Chimeric Antigen Receptor (CAR)-T Cells. Int. J. Mol. Sci..

[46] Jiang H., Shi Z., Wang P., Wang C., Yang L., Du G., Zhang H., Shi B., Jia J., Li Q. (2019). Claudin18.2-Specific Chimeric Antigen Receptor Engineered T Cells for the Treatment of Gastric Cancer. J. Natl. Cancer Inst..

[47] DeSelm C.J., Tano Z.E., Varghese A.M., Adusumilli P.S. (2017). CAR T-cell therapy for pancreatic cancer. J. Surg. Oncol..

[48] Staudt R.E., Carlson R.D., Snook A.E. (2022). Targeting gastrointestinal cancers with chimeric antigen receptor (CAR)-T cell therapy. Cancer Biol. Ther..

[49] Batra S.A., Rathi P., Guo L., Courtney A.N., Fleurence J., Balzeau J., Shaik R.S., Nguyen T.P., Wu M.-F., Bulsara S. (2020). Glypican-3-Specific CAR T Cells Coexpressing IL15 and IL21 Have Superior Expansion and Antitumor Activity against Hepatocellular Carcinoma. Cancer Immunol. Res..

[50] Wang X., Wong K., Ouyang W., Rutz S. (2019). Targeting IL-10 Family Cytokines for the Treatment of Human Diseases. Cold Spring Harb. Perspect. Biol..

[51] Neurath M.F. (2019). IL-23 in inflammatory bowel diseases and colon cancer. Cytokine Growth Factor Rev..

[52] Ouyang W., Rutz S., Crellin N.K., Valdez P.A., Hymowitz S.G. (2011). Regulation and functions of the IL-10 family of cytokines in inflammation and disease. Annu. Rev. Immunol..

[53] Saraiva M., Vieira P., O’Garra A. (2020). Biology and therapeutic potential of interleukin-10. J. Exp. Med..

[54] Franke A., Balschun T., Karlsen T.H., Sventoraityte J., Nikolaus S., Mayr G., Domingues F.S., Albrecht M., Nothnagel M., Ellinghaus D. (2008). Sequence variants in IL10, ARPC2 and multiple other loci contribute to ulcerative colitis susceptibility. Nat. Genet..

[55] Kühn R., Löhler J., Rennick D., Rajewsky K., Müller W. (1993). Interleukin-10-deficient mice develop chronic enterocolitis. Cell.

[56] Girardin S.E., Boneca I.G., Viala J., Chamaillard M., Labigne A., Thomas G., Philpott D.J., Sansonetti P.J. (2003). Nod2 is a general sensor of peptidoglycan through muramyl dipeptide (MDP) detection. J. Biol. Chem..

[57] DeWeerdt S. (2016). Genetics: Clues in the code. Nature.

[58] Lassen K.G., Kuballa P., Conway K.L., Patel K.K., Becker C.E., Peloquin J.M., Villablanca E.J., Norman J.M., Liu T.-C., Heath R.J. (2014). Atg16L1 T300A variant decreases selective autophagy resulting in altered cytokine signaling and decreased antibacterial defense. Proc. Natl. Acad. Sci. USA.

[59] Wei L., Ploss A. (2021). Mechanism of Hepatitis B Virus cccDNA Formation. Viruses.

[60] Weber N.D., Odriozola L., Martínez-García J., Ferrer V., Douar A., Bénichou B., González-Aseguinolaza G., Smerdou C. (2019). Gene therapy for progressive familial intrahepatic cholestasis type 3 in a clinically relevant mouse model. Nat. Commun..

[61] Kohut T.J., Gilbert M.A., Loomes K.M. (2021). Alagille Syndrome: A Focused Review on Clinical Features, Genetics, and Treatment. Semin. Liver Dis..

[62] Członkowska A., Litwin T., Dusek P., Ferenci P., Lutsenko S., Medici V., Rybakowski J.K., Weiss K.H., Schilsky M.L. (2018). Wilson disease. Nat. Rev. Dis. Primers.

[63] Strnad P., McElvaney N.G., Lomas D.A. (2020). Alpha1-Antitrypsin Deficiency. N. Engl. J. Med..

[64] Atchison R.W., Casto B.C., Hammon W.M. (1965). Adenovirus-associated defective virus particles. Science.

[65] Wang D., Tai P.W.L., Gao G. (2019). Adeno-associated virus vector as a platform for gene therapy delivery. Nat. Rev. Drug Discov..

[66] Naso M.F., Tomkowicz B., Perry W.L., Strohl W.R. (2017). Adeno-Associated Virus (AAV) as a Vector for Gene Therapy. BioDrugs..

[67] Lundstrom K. (2018). Viral Vectors in Gene Therapy. Diseases.

[68] Srivastava A. (2016). In vivo tissue-tropism of adeno-associated viral vectors. Curr. Opin. Virol..

[69] Kattenhorn L.M., Tipper C.H., Stoica L., Geraghty D.S., Wright T.L., Clark K.R., Wadsworth S.C. (2016). Adeno-Associated Virus Gene Therapy for Liver Disease. Hum. Gene Ther..

[70] Polyak S., Mah C., Porvasnik S., Herlihy J.-D., Campbell-Thompson M., Byrne B.J., Valentine J.F. (2008). Gene Delivery to Intestinal Epithelial Cells In vitro and In vivo with Recombinant Adeno-Associated Virus Types 1, 2 and 5. Dig. Dis. Sci..

[71] Ma L.-T., Lian J.-X., Bai Y., Shang M.-J., Zhang Z.-Z., Wu F.-F., Chen J., Meng X.-B., Zheng J., Li T. (2022). Adeno-associated virus vector intraperitoneal injection induces colonic mucosa and submucosa transduction and alters the diversity and composition of the faecal microbiota in rats. Front. Cell. Infect. Microbiol..

[72] Vilà L., Elias I., Roca C., Ribera A., Ferré T., Casellas A., Lage R., Franckhauser S., Bosch F. (2014). AAV8-mediated Sirt1 gene transfer to the liver prevents high carbohydrate diet-induced nonalcoholic fatty liver disease. Mol. Ther. Methods Clin. Dev..

[73] Ronzitti G., Gross D.-A., Mingozzi F. (2020). Human Immune Responses to Adeno-Associated Virus (AAV) Vectors. Front. Immunol..

[74] Dalwadi D.A., Torrens L., Abril-Fornaguera J., Pinyol R., Willoughby C., Posey J., Llovet J.M., Lanciault C., Russell D.W., Grompe M., Naugler W.E. (2021). Liver Injury Increases the Incidence of HCC following AAV Gene Therapy in Mice. Mol. Ther..

[75] Duan L., Xu L., Xu X., Qin Z., Zhou X., Xiao Y., Liang Y., Xia J. (2021). Exosome-mediated delivery of gene vectors for gene therapy. Nanoscale.

[76] Duan L., Ouyang K., Wang J., Xu L., Xu X., Wen C., Xie Y., Liang Y., Xia J. (2021). Exosomes as Targeted Delivery Platform of CRISPR/Cas9 for Therapeutic Genome Editing. Chembiochem..

[77] Liang Y., Iqbal Z., Lu J., Wang J., Zhang H., Chen X., Duan L., Xia J. (2023). Cell-Derived Nanovesicle-Mediated Drug Delivery to the Brain: Principles and Strategies for Vesicle Engineering. Mol. Ther..

[78] Liang Y., Xu X., Xu L., Iqbal Z., Ouyang K., Zhang H., Wen C., Duan L., Xia J. (2022). Chondrocyte-specific genomic editing enabled by hybrid exosomes for osteoarthritis treatment. Theranostics.

[79] Duan L., Xu X., Xu L., Chen H., Li X., Alahdal M., Xiao Y., Liang Y., Xia J. (2021). Exosome-Mediated Drug Delivery for Cell-Free Therapy of Osteoarthritis. Curr. Med. Chem..

[80] Liang Y., Duan L., Lu J., Xia J. (2021). Engineering exosomes for targeted drug delivery. Theranostics.

[81] Li D.-F., Yang M.-F., Xu J., Xu H.-M., Zhu M.-Z., Liang Y.-J., Zhang Y., Tian C.-M., Nie Y.-Q., Shi R.-Y. (2022). Extracellular Vesicles: The Next Generation Theranostic Nanomedicine for Inflammatory Bowel Disease. Int. J. Nanomed..

[82] Tian C.-M., Yang M.-F., Xu H.-M., Zhu M.-Z., Zhang Y., Yao J., Wang L.-S., Liang Y.-J., Li D.-F. (2023). Emerging role of bacterial outer membrane vesicle in gastrointestinal tract. Gut Pathog..

[83] Yin H., Kanasty R.L., Eltoukhy A.A., Vegas A.J., Dorkin J.R., Dg A., Anderson D.G. (2014). Non-viral vectors for gene-based therapy. Nat. Rev. Genet..

[84] Li D.-F., Liu Q.-S., Yang M.-F., Xu H.-M., Zhu M.-Z., Zhang Y., Xu J., Tian C.-M., Yao J., Wang L.-S., Liang Y.J. (2023). Nanomaterials for mRNA-based therapeutics: Challenges and opportunities. Bioeng. Transl. Med..

[85] Li D.-F., Yang M.-F., Xu H.-M., Zhu M.-Z., Zhang Y., Tian C.-M., Nie Y.-Q., Wang J.-Y., Liang Y.-J., Yao J., Wang L.S. (2022). Nanoparticles for oral delivery: targeted therapy for inflammatory bowel disease. J. Mater. Chem. B.

[86] Yue N.-N., Xu H.-M., Xu J., Zhu M.-Z., Zhang Y., Tian C.-M., Nie Y.-Q., Yao J., Liang Y.-J., Li D.-F., Wang L.S. (2023). Application of Nanoparticles in the Diagnosis of Gastrointestinal Diseases: A Complete Future Perspective. Int. J. Nanomed..

[87] Bangham A.D., Horne R.W. (1964). Negative staining of phospholipids and their structural modification by surface-active agents as observed in the electron microscope. J. Mol. Biol..

[88] Zu H., Gao D. (2021). Non-viral Vectors in Gene Therapy: Recent Development, Challenges, and Prospects. AAPS J..

[89] Gao X., Huang L. (1995). Cationic liposome-mediated gene transfer. Gene Ther..

[90] Karmali P.P., Chaudhuri A. (2007). Cationic liposomes as non-viral carriers of gene medicines: resolved issues, open questions, and future promises. Med. Res. Rev..

[91] Zhang R., Men K., Zhang X., Huang R., Tian Y., Zhou B., Yu C., Wang Y., Ji X., Hu Q., Yang L. (2018). Delivery of a Modified mRNA Encoding IL-22 Binding Protein (IL-22BP) for Colon Cancer Gene Therapy. J. Biomed. Nanotechnol..

[92] Peng Z., Wang C., Fang E., Lu X., Wang G., Tong Q. (2014). Co-delivery of doxorubicin and SATB1 shRNA by thermosensitive magnetic cationic liposomes for gastric cancer therapy. PLoS One.

[93] Samaridou E., Heyes J., Lutwyche P. (2020). Lipid nanoparticles for nucleic acid delivery: Current perspectives. Adv. Drug Deliv. Rev..

[94] Chen C.-K., Huang P.-K., Law W.-C., Chu C.-H., Chen N.-T., Lo L.-W. (2020). Biodegradable Polymers for Gene-Delivery Applications. Int. J. Nanomed..

[95] Zhao L., Li Y., Pei D., Huang Q., Zhang H., Yang Z., Li F., Shi T. (2019). Glycopolymers/PEI complexes as serum-tolerant vectors for enhanced gene delivery to hepatocytes. Carbohydr. Polym..

[96] Nishiyama N., Kataoka K. (2006). Current state, achievements, and future prospects of polymeric micelles as nanocarriers for drug and gene delivery. Pharmacol. Ther..

[97] Wang Y., Costanza F., Li C., Nimmagadda A., Song D., Cai J. (2016). PEG-Poly(amino acid)s/MicroRNA Complex Nanoparticles Effectively Arrest the Growth and Metastasis of Colorectal Cancer. J. Biomed. Nanotechnol..

[98] Loh X.J., Lee T.-C., Dou Q., Deen G.R. (2016). Utilising inorganic nanocarriers for gene delivery. Biomater. Sci..

[99] Panyala N.R., Peña-Méndez E.M., Havel J. (2009). Gold and nano-gold in medicine: overview, toxicology and perspectives. J. Appl. Biomed..

[100] Kafshdooz T., Kafshdooz L., Akbarzadeh A., Hanifehpour Y., Joo S.W. (2016). Applications of nanoparticle systems in gene delivery and gene therapy. Artif. Cells Nanomed. Biotechnol..

[101] Craciun B.F., Clima L., Bostiog D.-I., Silion M., Calin M., Peptanariu D., Pinteala M. (2023). Multilayer gold nanoparticles as non-viral vectors for targeting MCF-7 cancer cells. Biomater. Adv..

[102] Zhou Y., Quan G., Wu Q., Zhang X., Niu B., Wu B., Huang Y., Pan X., Wu C. (2018). Mesoporous silica nanoparticles for drug and gene delivery. Acta Pharm. Sin. B.

[103] Slowing I.I., Vivero-Escoto J.L., Wu C.-W., Lin V.S.-Y. (2008). Mesoporous silica nanoparticles as controlled release drug delivery and gene transfection carriers. Adv. Drug Deliv. Rev..

[104] Kim M.-H., Na H.-K., Kim Y.-K., Ryoo S.-R., Cho H.S., Lee K.E., Jeon H., Ryoo R., Min D.-H. (2011). Facile synthesis of monodispersed mesoporous silica nanoparticles with ultralarge pores and their application in gene delivery. ACS Nano.

[105] Tong S., Zhu H., Bao G. (2019). Magnetic Iron Oxide Nanoparticles for Disease Detection and Therapy. Mater. Today.

[106] Li H., Peng E., Zhao F., Li J., Xue J. (2021). Supramolecular Surface Functionalization of Iron Oxide Nanoparticles with α-Cyclodextrin-Based Cationic Star Polymer for Magnetically-Enhanced Gene Delivery. Pharmaceutics.

[107] Kim M.-C., Lin M.M., Sohn Y., Kim J.-J., Kang B.S., Kim D.K. (2017). Polyethyleneimine-associated polycaprolactone-Superparamagnetic iron oxide nanoparticles as a gene delivery vector. J. Biomed. Mater. Res. B Appl. Biomater..

[108] Cao H., Duan L., Zhang Y., Cao J., Zhang K. (2021). Current hydrogel advances in physicochemical and biological response-driven biomedical application diversity. Signal. Transduct. Target. Ther..

[109] Zhao D., Song H., Zhou X., Chen Y., Liu Q., Gao X., Zhu X., Chen D. (2019). Novel facile thermosensitive hydrogel as sustained and controllable gene release vehicle for breast cancer treatment. Eur. J. Pharm. Sci..

[110] Lan B., Zhang L., Yang L., Wu J., Li N., Pan C., Wang X., Zeng L., Yan L., Yang C., Ren M. (2021). Sustained delivery of MMP-9 siRNA via thermosensitive hydrogel accelerates diabetic wound healing. J. Nanobiotechnol..

[111] Kim Y.-M., Song S.-C. (2014). Targetable micelleplex hydrogel for long-term, effective, and systemic siRNA delivery. Biomaterials.

[112] Youngblood R.L., Truong N.F., Segura T., Shea L.D. (2018). It’s All in the Delivery: Designing Hydrogels for Cell and Non-viral Gene Therapies. Mol. Ther..

[113] Zhao B., Zhou B., Shi K., Zhang R., Dong C., Xie D., Tang L., Tian Y., Qian Z., Yang L. (2021). Sustained and targeted delivery of siRNA/DP7-C nanoparticles from injectable thermosensitive hydrogel for hepatocellular carcinoma therapy. Cancer Sci..

[114] Agrawal M., Allin K.H., Petralia F., Colombel J.-F., Jess T. (2022). Multiomics to elucidate inflammatory bowel disease risk factors and pathways. Nat. Rev. Gastroenterol. Hepatol..

[115] Liu J.Z., van Sommeren S., Huang H., Ng S.C., Alberts R., Takahashi A., Ripke S., Lee J.C., Jostins L., Shah T. (2015). Association analyses identify 38 susceptibility loci for inflammatory bowel disease and highlight shared genetic risk across populations. Nat. Genet..

[116] Ackermann M., Mucci A., McCabe A., Frei S., Wright K., Snapper S.B., Lachmann N., Williams D.A., Brendel C. (2021). Restored Macrophage Function Ameliorates Disease Pathophysiology in a Mouse Model for IL10 Receptor-deficient Very Early Onset Inflammatory Bowel Disease. J. Crohns Colitis.

[117] Sasaki M., Mathis J.M., Jennings M.H., Jordan P., Wang Y., Ando T., Joh T., Alexander J.S. (2005). Reversal of experimental colitis disease activity in mice following administration of an adenoviral IL-10 vector. J. Inflamm..

[118] Sugimoto K., Ogawa A., Mizoguchi E., Shimomura Y., Andoh A., Bhan A.K., Blumberg R.S., Xavier R.J., Mizoguchi A. (2008). IL-22 ameliorates intestinal inflammation in a mouse model of ulcerative colitis. J. Clin. Invest..

[119] Zuo L., Huang Z., Dong L., Xu L., Zhu Y., Zeng K., Zhang C., Chen J., Zhang J. (2010). Targeting delivery of anti-TNFalpha oligonucleotide into activated colonic macrophages protects against experimental colitis. Gut.

[120] Song Y., Kim Y.-R., Kim S.M., Ul Ain Q., Jang K., Yang C.-S., Kim Y.-H. (2016). RNAi-mediated silencing of TNF-α converting enzyme to down-regulate soluble TNF-α production for treatment of acute and chronic colitis. J. Control Release..

[121] Xiao B., Chen Q., Zhang Z., Wang L., Kang Y., Denning T., Merlin D. (2018). TNFα gene silencing mediated by orally targeted nanoparticles combined with interleukin-22 for synergistic combination therapy of ulcerative colitis. J. Control Release..

[122] Nold M.F., Nold-Petry C.A., Zepp J.A., Palmer B.E., Bufler P., Dinarello C.A. (2010). IL-37 is a fundamental inhibitor of innate immunity. Nat. Immunol..

[123] Wang W.q., Dong K., Zhou L., Jiao G.h., Zhu C.z., Li W.w., Yu G., Wu W.t., Chen S., Sun Z.n. (2015). IL-37b gene transfer enhances the therapeutic efficacy of mesenchumal stromal cells in DSS-induced colitis mice. Acta Pharmacol. Sin..

[124] Nowarski R., Jackson R., Gagliani N., de Zoete M.R., Palm N.W., Bailis W., Low J.S., Harman C.C.D., Graham M., Elinav E., Flavell R.A. (2015). Epithelial IL-18 Equilibrium Controls Barrier Function in Colitis. Cell.

[125] Prieto J., Herraiz M., Sangro B., Qian C., Mazzolini G., Melero I., Ruiz J. (2003). The promise of gene therapy in gastrointestinal and liver diseases. Gut.

[126] Hagemeyer N., Kierdorf K., Frenzel K., Xue J., Ringelhan M., Abdullah Z., Godin I., Wieghofer P., Costa Jordão M.J., Ulas T. (2016). Transcriptome-based profiling of yolk sac-derived macrophages reveals a role for Irf8 in macrophage maturation. EMBO J..

[127] Kurotaki D., Osato N., Nishiyama A., Yamamoto M., Ban T., Sato H., Nakabayashi J., Umehara M., Miyake N., Matsumoto N. (2013). Essential role of the IRF8-KLF4 transcription factor cascade in murine monocyte differentiation. Blood.

[128] Ramos P.S., Shedlock A.M., Langefeld C.D. (2015). Genetics of autoimmune diseases: insights from population genetics. J. Hum. Genet..

[129] Veiga N., Goldsmith M., Diesendruck Y., Ramishetti S., Rosenblum D., Elinav E., Behlke M.A., Benhar I., Peer D. (2019). Leukocyte-specific siRNA delivery revealing IRF8 as a potential anti-inflammatory target. J. Control Release..

[130] Nata T., Fujiya M., Ueno N., Moriichi K., Konishi H., Tanabe H., Ohtake T., Ikuta K., Kohgo Y. (2013). MicroRNA-146b improves intestinal injury in mouse colitis by activating nuclear factor-κB and improving epithelial barrier function. J. Gene Med..

[131] Yan X., Pan Q., Xin H., Chen Y., Ping Y. (2021). Genome-editing prodrug: Targeted delivery and conditional stabilization of CRISPR-Cas9 for precision therapy of inflammatory disease. Sci. Adv..

[132] Arnold M., Abnet C.C., Neale R.E., Vignat J., Giovannucci E.L., McGlynn K.A., Bray F. (2020). Global Burden of 5 Major Types of Gastrointestinal Cancer. Gastroenterology.

[133] Song X., Liu C., Wang N., Huang H., He S., Gong C., Wei Y. (2021). Delivery of CRISPR/Cas systems for cancer gene therapy and immunotherapy. Adv. Drug Deliv. Rev..

[134] Navarro S.A., Carrillo E., Griñán-Lisón C., Martín A., Perán M., Marchal J.A., Boulaiz H. (2016). Cancer suicide gene therapy: a patent review. Expert Opin. Ther. Pat..

[135] Cui H., Shen X., Chen D. (2015). Combined chemotherapy and gene therapy of esophageal cancer with human adenoviral p53 administered by endoscopic injection combined with chemotherapy. J. Clin. Oncol..

[136] Dahia P.L. (2000). PTEN, a unique tumor suppressor gene. Endocr. Relat. Cancer.

[137] Ge M.-K., Zhang N., Xia L., Zhang C., Dong S.-S., Li Z.-M., Ji Y., Zheng M.-H., Sun J., Chen G.-Q., Shen S.M. (2020). FBXO22 degrades nuclear PTEN to promote tumorigenesis. Nat. Commun..

[138] Niu L.-J., Huang T., Wang L., Sun X.-F., Zhang Y.-M. (2022). HBX suppresses PTEN to promote the malignant progression of hepatocellular carcinoma through mi-R155 activation. Ann. Hepatol..

[139] Xiao S., Liu Z., Deng R., Li C., Fu S., Chen G., Zhang X., Ke F., Ke S., Yu X. (2017). Aptamer-mediated gene therapy enhanced antitumor activity against human hepatocellular carcinoma in vitro and in vivo. J. Control Release..

[140] Zhang H., Zhou X., Xu C., Yang J., Xiang J., Tao M., Xie Y. (2016). Synergistic tumor suppression by adenovirus-mediated ING4/PTEN double gene therapy for gastric cancer. Cancer Gene Ther..

[141] Peer D., Lieberman J. (2011). Special delivery: targeted therapy with small RNAs. Gene Ther..

[142] Yang C., Hu R., Anderson T., Wang Y., Lin G., Law W.-C., Lin W.-J., Nguyen Q.T., Toh H.T., Yoon H.S. (2015). Biodegradable nanoparticle-mediated K-ras down regulation for pancreatic cancer gene therapy. J. Mater. Chem. B.

[143] Reghupaty S.C., Sarkar D. (2019). Current Status of Gene Therapy in Hepatocellular Carcinoma. Cancers.

[144] Liu S.-X., Xia Z.-S., Zhong Y.-Q. (2014). Gene therapy in pancreatic cancer. World J. Gastroenterol..

[145] Qu L., Wang Y., Gong L., Zhu J., Gong R., Si J. (2013). Suicide gene therapy for hepatocellular carcinoma cells by survivin promoter-driven expression of the herpes simplex virus thymidine kinase gene. Oncol. Rep..

[146] Wang Z., Chang Z., Lu M., Shao D., Yue J., Yang D., Zheng X., Li M., He K., Zhang M. (2018). Shape-controlled magnetic mesoporous silica nanoparticles for magnetically-mediated suicide gene therapy of hepatocellular carcinoma. Biomaterials.

[147] Hiraoka K., Kimura T., Logg C.R., Tai C.-K., Haga K., Lawson G.W., Kasahara N. (2007). Therapeutic efficacy of replication-competent retrovirus vector-mediated suicide gene therapy in a multifocal colorectal cancer metastasis model. Cancer Res..

[148] Ahn Y.-H., Yi H., Shin J.-Y., Lee K.-D., Shin S.-P., Lee S.-J., Song J., Chun K.-H. (2012). STAT3 silencing enhances the efficacy of the HSV.tk suicide gene in gastrointestinal cancer therapy. Clin. Exp. Metastasis.

[149] Qiu N., Wang G., Wang J., Zhou Q., Guo M., Wang Y., Hu X., Zhou H., Bai R., You M. (2021). Tumor-Associated Macrophage and Tumor-Cell Dually Transfecting Polyplexes for Efficient Interleukin-12 Cancer Gene Therapy. Adv. Mater..

[150] Chen Y., E C.-Y., Gong Z.-W., Liu S., Wang Z.-X., Yang Y.-S., Zhang X.-W. (2018). Chimeric antigen receptor-engineered T-cell therapy for liver cancer. Hepatobiliary Pancreat. Dis. Int..

[151] Gatti-Mays M.E., Redman J.M., Collins J.M., Bilusic M. (2017). Cancer vaccines: Enhanced immunogenic modulation through therapeutic combinations. Hum. Vaccin. Immunother..

[152] June C.H., O’Connor R.S., Kawalekar O.U., Ghassemi S., Milone M.C. (2018). CAR T cell immunotherapy for human cancer. Science.

[153] Leidner R., Sanjuan Silva N., Huang H., Sprott D., Zheng C., Shih Y.-P., Leung A., Payne R., Sutcliffe K., Cramer J. (2022). Neoantigen T-Cell Receptor Gene Therapy in Pancreatic Cancer. N. Engl. J. Med..

[154] Dimitri A., Herbst F., Fraietta J.A. (2022). Engineering the next-generation of CAR T-cells with CRISPR-Cas9 gene editing. Mol. Cancer.

[155] Newick K., O’Brien S., Moon E., Albelda S.M. (2017). CAR T Cell Therapy for Solid Tumors. Annu. Rev. Med..

[156] Chmielewski M., Hombach A.A., Abken H. (2014). Of CARs and TRUCKs: chimeric antigen receptor (CAR) T cells engineered with an inducible cytokine to modulate the tumor stroma. Immunol. Rev..

[157] Newick K., O’Brien S., Sun J., Kapoor V., Maceyko S., Lo A., Puré E., Moon E., Albelda S.M. (2016). Augmentation of CAR T-cell Trafficking and Antitumor Efficacy by Blocking Protein Kinase A Localization. Cancer Immunol. Res..

[158] Siveen K.S., Prabhu K., Krishnankutty R., Kuttikrishnan S., Tsakou M., Alali F.Q., Dermime S., Mohammad R.M., Uddin S. (2017). Vascular Endothelial Growth Factor (VEGF) Signaling in Tumour Vascularization: Potential and Challenges. Curr. Vasc. Pharmacol..

[159] Wang G., Gao X., Gu G., Shao Z., Li M., Wang P., Yang J., Cai X., Li Y. (2017). Polyethylene glycol-poly(ε-benzyloxycarbonyl-l-lysine)-conjugated VEGF siRNA for antiangiogenic gene therapy in hepatocellular carcinoma. Int. J. Nanomed..

[160] Lin D., Shen Y., Liang T. (2023). Oncolytic virotherapy: basic principles, recent advances and future directions. Signal. Transduct. Target. Ther..

[161] Tian Y., Xie D., Yang L. (2022). Engineering strategies to enhance oncolytic viruses in cancer immunotherapy. Signal. Transduct. Target. Ther..

[162] Park B.-H., Hwang T., Liu T.-C., Sze D.Y., Kim J.-S., Kwon H.-C., Oh S.Y., Han S.-Y., Yoon J.-H., Hong S.-H. (2008). Use of a targeted oncolytic poxvirus, JX-594, in patients with refractory primary or metastatic liver cancer: a phase I trial. Lancet Oncol..

[163] Heo J., Reid T., Ruo L., Breitbach C.J., Rose S., Bloomston M., Cho M., Lim H.Y., Chung H.C., Kim C.W. (2013). Randomized dose-finding clinical trial of oncolytic immunotherapeutic vaccinia JX-594 in liver cancer. Nat. Med..

[164] Bazan-Peregrino M., Garcia-Carbonero R., Laquente B., Álvarez R., Mato-Berciano A., Gimenez-Alejandre M., Morgado S., Rodríguez-García A., Maliandi M.V., Riesco M.C. (2021). VCN-01 disrupts pancreatic cancer stroma and exerts antitumor effects. J. Immunother. Cancer.

[165] Huang L., Zhao H., Shan M., Chen H., Xu B., He Y., Zhao Y., Liu Z., Chen J., Xu Q. (2022). Oncolytic adenovirus H101 ameliorate the efficacy of anti-PD-1 monotherapy in colorectal cancer. Cancer Med..

[166] Ma R., Li Z., Chiocca E.A., Caligiuri M.A., Yu J. (2023). The emerging field of oncolytic virus-based cancer immunotherapy. Trends Cancer.

[167] Glue P., Rouzier-Panis R., Raffanel C., Sabo R., Gupta S.K., Salfi M., Jacobs S., Clement R.P. (2000). A dose-ranging study of pegylated interferon alfa-2b and ribavirin in chronic hepatitis C. The Hepatitis C Intervention Therapy Group. Hepatol. Baltim. Md..

[168] Chien R.-N., Liaw Y.-F. (2022). Current Trend in Antiviral Therapy for Chronic Hepatitis B. Viruses.

[169] Guo J.-T., Guo H. (2015). Metabolism and function of hepatitis B virus cccDNA: Implications for the development of cccDNA-targeting antiviral therapeutics. Antivir. Res..

[170] Lin S.-R., Yang H.-C., Kuo Y.-T., Liu C.-J., Yang T.-Y., Sung K.-C., Lin Y.-Y., Wang H.-Y., Wang C.-C., Shen Y.-C. (2014). The CRISPR/Cas9 System Facilitates Clearance of the Intrahepatic HBV Templates In Vivo. Mol. Ther. Nucleic Acids.

[171] Ely A., Arbuthnot P. (2015). Differing prospects for the future of using gene therapy to treat infections with hepatitis B virus and hepatitis C virus. Discov. Med..

[172] Verstegen M.M.A., Pan Q., van der Laan L.J.W. (2015). Gene therapies for hepatitis C virus. Adv. Exp. Med. Biol..

[173] Pan Q., Ramakrishnaiah V., Henry S., Fouraschen S., de Ruiter P.E., Kwekkeboom J., Tilanus H.W., Janssen H.L.A., van der Laan L.J.W. (2012). Hepatic cell-to-cell transmission of small silencing RNA can extend the therapeutic reach of RNA interference (RNAi). Gut.

[174] Khaliq S., Jahan S., Ijaz B., Ahmad W., Asad S., Pervaiz A., Samreen B., Khan M., Hassan S. (2010). Inhibition of core gene of HCV 3a genotype using synthetic and vector derived siRNAs. Virol. J..

[175] Khaliq S., Jahan S., Pervaiz A., Ali Ashfaq U., Hassan S. (2011). Down-regulation of IRES containing 5’UTR of HCV genotype 3a using siRNAs. Virol. J..

[176] Panigrahi M., Thibault P.A., Wilson J.A. (2022). MicroRNA 122 Affects both the Initiation and the Maintenance of Hepatitis C Virus Infections. J. Virol..

[177] Fu H., Zhang X., Wang Q., Sun Y., Liu L., Huang L., Ding L., Shen M., Zhang L., Duan Y. (2018). Simple and rational design of a polymer nano-platform for high performance of HCV related miR-122 reduction in the liver. Biomater. Sci..

[178] Siew S.M., Cunningham S.C., Zhu E., Tay S.S., Venuti E., Bolitho C., Alexander I.E. (2019). Prevention of Cholestatic Liver Disease and Reduced Tumorigenicity in a Murine Model of PFIC Type 3 Using Hybrid AAV-piggyBac Gene Therapy. Hepatology.

[179] Aronson S.J., Bakker R.S., Shi X., Duijst S., Ten Bloemendaal L., de Waart D.R., Verheij J., Ronzitti G., Oude Elferink R.P., Beuers U. (2019). Liver-directed gene therapy results in long-term correction of progressive familial intrahepatic cholestasis type 3 in mice. J. Hepatol..

[180] Jones M.K., Kawanaka H., Baatar D., Szabó I.L., Tsugawa K., Pai R., Koh G.Y., Kim I., Sarfeh I.J., Tarnawski A.S. (2001). Gene therapy for gastric ulcers with single local injection of naked DNA encoding VEGF and angiopoietin-1. Gastroenterology.

[181] Szabo S., Deng X., Tolstanova G., Khomenko T., Paunovic B., Chen L., Jadus M., Sandor Z. (2011). Angiogenic and anti-angiogenic therapy for gastrointestinal ulcers: new challenges for rational therapeutic predictions and drug design. Curr. Pharm. Des..

[182] Lee P.J., Papachristou G.I. (2019). New insights into acute pancreatitis. Nat. Rev. Gastroenterol. Hepatol..

[183] Miao B., Qi W.-J., Zhang S.-W., Wang H., Wang C., Hu L., Huang G.-W., Li S.-R., Wang H. (2019). miR-148a suppresses autophagy by down-regulation of IL-6/STAT3 signaling in cerulein-induced acute pancreatitis. Pancreatology.

[184] Rodriguez-Nicolas A., Martínez-Chamorro A., Jiménez P., Matas-Cobos A.M., Redondo-Cerezo E., Ruiz-Cabello F. (2018). TH1 and TH2 Cytokine Profiles as Predictors of Severity in Acute Pancreatitis. Pancreas.

[185] Zou W.-G., Wang D.-S., Lang M.-F., Jin D.-Y., Xu D.-H., Zheng Z.-C., Wu Z.-H., Liu X.-Y. (2002). Human interleukin 10 gene therapy decreases the severity and mortality of lethal pancreatitis in rats. J. Surg. Res..

[186] Tan J.-H., Cao R.-C., Zhou L., Zhou Z.-T., Chen H.-J., Xu J., Chen X.-M., Jin Y.-C., Lin J.-Y., Zeng J.-L. (2020). ATF6 aggravates acinar cell apoptosis and injury by regulating p53/AIFM2 transcription in Severe Acute Pancreatitis. Theranostics.

[187] Westlund K.N. (2009). Gene therapy for pancreatitis pain. Gene Ther..

[188] Gordon E.M., Cornelio G.H., Lorenzo C.C., Levy J.P., Reed R.A., Liu L., Hall F.L. (2004). First clinical experience using a “pathotropic” injectable retroviral vector (Rexin-G) as intervention for stage IV pancreatic cancer. Int. J. Oncol..

[189] Gastric Cancer CAR T-cell Target Antigen ID’d (2021). Cancer Discov. 11, 2954. 10.1158/2159-8290.CD-NB2021-0390. 34642170

[190] Hege K.M., Bergsland E.K., Fisher G.A., Nemunaitis J.J., Warren R.S., McArthur J.G., Lin A.A., Schlom J., June C.H., Sherwin S.A. (2017). Safety, tumor trafficking and immunogenicity of chimeric antigen receptor (CAR)-T cells specific for TAG-72 in colorectal cancer. J. Immunother. Cancer.

[191] Qi C., Gong J., Li J., Liu D., Qin Y., Ge S., Zhang M., Peng Z., Zhou J., Cao Y. (2022). Claudin18.2-specific CAR T cells in gastrointestinal cancers: phase 1 trial interim results. Nat. Med..

[192] Rogy M.A., Beinhauer B.G., Reinisch W., Huang L., Pokieser P. (2000). Transfer of interleukin-4 and interleukin-10 in patients with severe inflammatory bowel disease of the rectum. Hum. Gene Ther..

[193] Braat H., Rottiers P., Hommes D.W., Huyghebaert N., Remaut E., Remon J.-P., van Deventer S.J.H., Neirynck S., Peppelenbosch M.P., Steidler L. (2006). A phase I trial with transgenic bacteria expressing interleukin-10 in Crohn’s disease. Clin. Gastroenterol. Hepatol..

[194] Kanda M., Shimizu D., Tanaka H., Tanaka C., Kobayashi D., Hayashi M., Takami H., Niwa Y., Yamada S., Fujii T. (2018). Synaptotagmin XIII expression and peritoneal metastasis in gastric cancer. Br. J. Surg..

[195] Lei S., Zhang X., Men K., Gao Y., Yang X., Wu S., Duan X., Wei Y., Tong R. (2020). Efficient Colorectal Cancer Gene Therapy with IL-15 mRNA Nanoformulation. Mol. Pharm..

[196] O’Neill M.J., Bourre L., Melgar S., O’Driscoll C.M. (2011). Intestinal delivery of non-viral gene therapeutics: physiological barriers and preclinical models. Drug Discov. Today.

[197] La Bella T., Imbeaud S., Peneau C., Mami I., Datta S., Bayard Q., Caruso S., Hirsch T.Z., Calderaro J., Morcrette G. (2020). Adeno-associated virus in the liver: natural history and consequences in tumour development. Gut.

[198] Veiga N., Goldsmith M., Granot Y., Rosenblum D., Dammes N., Kedmi R., Ramishetti S., Peer D. (2018). Cell specific delivery of modified mRNA expressing therapeutic proteins to leukocytes. Nat. Commun..

[199] Peer D., Park E.J., Morishita Y., Carman C.V., Shimaoka M. (2008). Systemic leukocyte-directed siRNA delivery revealing cyclin D1 as an anti-inflammatory target. Science.

[200] Xu X., Liu P., Yuan Z., Wang D., Lu Q., Zhang Z., Jiang Q., Shi D., Yin L. (2019). Efficient and targeted drug/siRNA co-delivery mediated by reversibly crosslinked polymersomes toward anti-inflammatory treatment of ulcerative colitis (UC). Nano Res..

[201] Frede A., Neuhaus B., Klopfleisch R., Walker C., Buer J., Müller W., Epple M., Westendorf A.M. (2016). Colonic gene silencing using siRNA-loaded calcium phosphate/PLGA nanoparticles ameliorates intestinal inflammation in vivo. J. Control Release..

[202] Laroui H., Geem D., Xiao B., Viennois E., Rakhya P., Denning T., Merlin D. (2014). Targeting intestinal inflammation with CD98 siRNA/PEI-loaded nanoparticles. Mol. Ther..

[203] Xiao B., Laroui H., Viennois E., Ayyadurai S., Charania M.A., Zhang Y., Zhang Z., Baker M.T., Zhang B., Gewirtz A.T., Merlin D. (2014). Nanoparticles with surface antibody against CD98 and carrying CD98 small interfering RNA reduce colitis in mice. Gastroenterology.

[204] Laroui H., Viennois E., Xiao B., Canup B.S.B., Geem D., Denning T.L., Merlin D. (2014). Fab’-bearing siRNA TNFα-loaded nanoparticles targeted to colonic macrophages offer an effective therapy for experimental colitis. J. Control Release..

[205] Huang Y., Guo J., Gui S. (2018). Orally targeted galactosylated chitosan poly(lactic-co-glycolic acid) nanoparticles loaded with TNF-ɑ siRNA provide a novel strategy for the experimental treatment of ulcerative colitis. Eur. J. Pharm. Sci..

[206] Xiao B., Zhang Z., Viennois E., Kang Y., Zhang M., Han M.K., Chen J., Merlin D. (2016). Combination Therapy for Ulcerative Colitis: Orally Targeted Nanoparticles Prevent Mucosal Damage and Relieve Inflammation. Theranostics.

[207] Chu S., Tang C., Yin C. (2015). Effects of mannose density on in vitro and in vivo cellular uptake and RNAi efficiency of polymeric nanoparticles. Biomaterials.

[208] Zhang J., Tang C., Yin C. (2013). Galactosylated trimethyl chitosan-cysteine nanoparticles loaded with Map4k4 siRNA for targeting activated macrophages. Biomaterials.

[209] Bao W.-L., Wu Q., Hu B., Sun D., Zhao S., Shen X., Cheng H., Shen W. (2021). Oral Nanoparticles of SNX10-shRNA Plasmids Ameliorate Mouse Colitis. Int. J. Nanomed..

[210] Xiao B., Laroui H., Ayyadurai S., Viennois E., Charania M.A., Zhang Y., Merlin D. (2013). Mannosylated bioreducible nanoparticle-mediated macrophage-specific TNF-α RNA interference for IBD therapy. Biomaterials.

[211] Knipe J.M., Strong L.E., Peppas N.A. (2016). Enzyme- and pH-Responsive Microencapsulated Nanogels for Oral Delivery of siRNA to Induce TNF-α Knockdown in the Intestine. Biomacromolecules.

[212] Wilson D.S., Dalmasso G., Wang L., Sitaraman S.V., Merlin D., Murthy N. (2010). Orally delivered thioketal nanoparticles loaded with TNF-α-siRNA target inflammation and inhibit gene expression in the intestines. Nat. Mater..

[213] Zhang S., Dong Y., Wang Y., Sun W., Wei M., Yuan L., Yang G. (2021). Selective Encapsulation of Therapeutic mRNA in Engineered Extracellular Vesicles by DNA Aptamer. Nano Lett..

[214] Jun Y., Tang Z., Luo C., Jiang B., Li X., Tao M., Gu H., Liu L., Zhang Z., Sun S. (2020). Leukocyte-Mediated Combined Targeted Chemo and Gene Therapy for Esophageal Cancer. ACS Appl. Mater. Inter..

[215] Wan T., Pan Q., Liu C., Guo J., Li B., Yan X., Cheng Y., Ping Y. (2021). A Duplex CRISPR-Cas9 Ribonucleoprotein Nanomedicine for Colorectal Cancer Gene Therapy. Nano Lett..

[216] Li Y., Zhao Z., Lin C.-Y., Liu Y., Staveley-OCarroll K.F., Li G., Cheng K. (2021). Silencing PCBP2 normalizes desmoplastic stroma and improves the antitumor activity of chemotherapy in pancreatic cancer. Theranostics.

[217] Wan T., Chen Y., Pan Q., Xu X., Kang Y., Gao X., Huang F., Wu C., Ping Y. (2020). Genome editing of mutant KRAS through supramolecular polymer-mediated delivery of Cas9 ribonucleoprotein for colorectal cancer therapy. J. Control Release..

[218] Zheng Q.C., Jiang S., Wu Y.Z., Shang D., Zhang Y., Hu S.B., Cheng X., Zhang C., Sun P., Gao Y. (2020). Dual-Targeting Nanoparticle-Mediated Gene Therapy Strategy for Hepatocellular Carcinoma by Delivering Small Interfering RNA. Front. Bioeng. Biotechnol..

[219] Kamimura K., Yokoo T., Abe H., Sakai N., Nagoya T., Kobayashi Y., Ohtsuka M., Miura H., Sakamaki A., Kamimura H. (2020). Effect of Diphtheria Toxin-Based Gene Therapy for Hepatocellular Carcinoma. Cancers.

[220] Yoo B., Jordan V.C., Sheedy P., Billig A.-M., Ross A., Pantazopoulos P., Medarova Z. (2019). RNAi-Mediated PD-L1 Inhibition for Pancreatic Cancer Immunotherapy. Sci. Rep..

[221] Zhao X., Wang X., Sun W., Cheng K., Qin H., Han X., Lin Y., Wang Y., Lang J., Zhao R. (2018). Precision design of nanomedicines to restore gemcitabine chemosensitivity for personalized pancreatic ductal adenocarcinoma treatment. Biomaterials.

[222] Yin F., Hu K., Chen Y., Yu M., Wang D., Wang Q., Yong K.-T., Lu F., Liang Y., Li Z. (2017). SiRNA Delivery with PEGylated Graphene Oxide Nanosheets for Combined Photothermal and Genetherapy for Pancreatic Cancer. Theranostics.

[223] Ju H.-Q., Lu Y.-X., Wu Q.-N., Liu J., Zeng Z.-L., Mo H.-Y., Chen Y., Tian T., Wang Y., Kang T.-B. (2017). Disrupting G6PD-mediated Redox homeostasis enhances chemosensitivity in colorectal cancer. Oncogene.

[224] Liu X., Gao X., Zheng S., Wang B., Li Y., Zhao C., Muftuoglu Y., Chen S., Li Y., Yao H. (2017). Modified nanoparticle mediated IL-12 immunogene therapy for colon cancer. Nanomedicine..

[225] Li Y., Chen Y., Li J., Zhang Z., Huang C., Lian G., Yang K., Chen S., Lin Y., Wang L. (2017). Co-delivery of microRNA-21 antisense oligonucleotides and gemcitabine using nanomedicine for pancreatic cancer therapy. Cancer Sci..

[226] Liu T., Wu H.-J., Liang Y., Liang X.-J., Huang H.-C., Zhao Y.-Z., Liao Q.-C., Chen Y.-Q., Leng A.-M., Yuan W.-J. (2016). Tumor-specific expression of shVEGF and suicide gene as a novel strategy for esophageal cancer therapy. World J. Gastroenterol..

[227] Zhao X., Li F., Li Y., Wang H., Ren H., Chen J., Nie G., Hao J. (2015). Co-delivery of HIF1α siRNA and gemcitabine via biocompatible lipid-polymer hybrid nanoparticles for effective treatment of pancreatic cancer. Biomaterials.

[228] Wu Y., Guo Z., Zhang D., Zhang W., Yan Q., Shi X., Zhang M., Zhao Y., Zhang Y., Jiang B. (2013). A novel colon cancer gene therapy using rAAV-mediated expression of human shRNA-FHL2. Int. J. Oncol..

[229] Aguilar L.K., Shirley L.A., Chung V.M., Marsh C.L., Walker J., Coyle W., Marx H., Bekaii-Saab T., Lesinski G.B., Swanson B. (2015). Gene-mediated cytotoxic immunotherapy as adjuvant to surgery or chemoradiation for pancreatic adenocarcinoma. Cancer Immunol. Immunother..

[230] Buscail L., Bournet B., Vernejoul F., Cambois G., Lulka H., Hanoun N., Dufresne M., Meulle A., Vignolle-Vidoni A., Ligat L. (2015). First-in-man phase 1 clinical trial of gene therapy for advanced pancreatic cancer: safety, biodistribution, and preliminary clinical findings. Mol. Ther..

[231] Le D.T., Wang-Gillam A., Picozzi V., Greten T.F., Crocenzi T., Springett G., Morse M., Zeh H., Cohen D., Fine R.L. (2015). Safety and survival with GVAX pancreas prime and Listeria Monocytogenes-expressing mesothelin (CRS-207) boost vaccines for metastatic pancreatic cancer. J. Clin. Oncol..

[232] Noonan A.M., Farren M.R., Geyer S.M., Huang Y., Tahiri S., Ahn D., Mikhail S., Ciombor K.K., Pant S., Aparo S. (2016). Randomized Phase 2 Trial of the Oncolytic Virus Pelareorep (Reolysin) in Upfront Treatment of Metastatic Pancreatic Adenocarcinoma. Mol. Ther..

[233] Lee J.-C., Shin D.W., Park H., Kim J., Youn Y., Kim J.H., Kim J., Hwang J.-H. (2020). Tolerability and safety of EUS-injected adenovirus-mediated double-suicide gene therapy with chemotherapy in locally advanced pancreatic cancer: a phase 1 trial. Gastrointest. Endosc..

[234] Sangro B., Mazzolini G., Ruiz M., Ruiz J., Quiroga J., Herrero I., Qian C., Benito A., Larrache J., Olagüe C. (2010). A phase I clinical trial of thymidine kinase-based gene therapy in advanced hepatocellular carcinoma. Cancer Gene Ther..

[235] Chang K.J., Reid T., Senzer N., Swisher S., Pinto H., Hanna N., Chak A., Soetikno R. (2012). Phase I evaluation of TNFerade biologic plus chemoradiotherapy before esophagectomy for locally advanced resectable esophageal cancer. Gastrointest. Endosc..

[236] Peng K., Qian X., Huang Z., Lu J., Wang Y., Zhou Y., Wang H., Wu B., Wang Y., Chen L. (2018). Umbilical Cord Blood Transplantation Corrects Very Early-Onset Inflammatory Bowel Disease in Chinese Patients With IL10RA-Associated Immune Deficiency. Inflamm. Bowel Dis..

